# Impact of a 2-year trial of nutritional ketosis on indices of cardiovascular disease risk in patients with type 2 diabetes

**DOI:** 10.1186/s12933-020-01178-2

**Published:** 2020-12-08

**Authors:** Shaminie J. Athinarayanan, Sarah J. Hallberg, Amy L. McKenzie, Katharina Lechner, Sarah King, James P. McCarter, Jeff S. Volek, Stephen D. Phinney, Ronald M. Krauss

**Affiliations:** 1 Virta Health, 501 Folsom Street, San Francisco, CA 94105 USA; 2 Indiana University Health Arnett, Lafayette, IN USA; 3 Indiana University, School of Medicine, Indianapolis, IN USA; 4 Department of Cardiology, German Heart Centre Munich, Technical University Munich, Munich, Germany; 5 DZHK (German Centre for Cardiovascular Research), Partner Site Munich, Munich Heart Alliance, Munich, Germany; 6 School of Medicine, University of California, San Francisco, CA 94143 USA; 7 Abbott Diabetes Care, Alameda, CA 94502 USA; 8 Department of Genetics, Washington University School of Medicine, St. Louis, MO USA; 9 Department of Human Sciences, The Ohio State University, Columbus, OH USA

**Keywords:** Type 2 diabetes, Nutritional ketosis, Cardiovascular risk, Lipoprotein sub-fractionation, Atherogenic lipoprotein phenotype

## Abstract

**Background:**

We have previously reported that in patients with type 2 diabetes (T2D) consumption of a very low carbohydrate diet capable of inducing nutritional ketosis over 2 years (continuous care intervention, CCI) resulted in improved body weight, glycemic control, and multiple risk factors for cardiovascular disease (CVD) with the exception of an increase in low density lipoprotein cholesterol (LDL-C). In the present study, we report the impact of this intervention on markers of risk for atherosclerotic cardiovascular disease (CVD), with a focus on lipoprotein subfraction particle concentrations as well as carotid-artery intima-media thickness (CIMT).

**Methods:**

Analyses were performed in patients with T2D who completed 2 years of this study (CCI; n = 194; usual care (UC): n = 68). Lipoprotein subfraction particle concentrations were measured by ion mobility at baseline, 1, and 2 years and CIMT was measured at baseline and 2 years. Principal component analysis (PCA) was used to assess changes in independent clusters of lipoprotein particles.

**Results:**

At 2 years, CCI resulted in a 23% decrease of small LDL IIIb and a 29% increase of large LDL I with no change in total LDL particle concentration or ApoB. The change in proportion of smaller and larger LDL was reflected by reversal of the small LDL subclass phenotype B in a high proportion of CCI participants (48.1%) and a shift in the principal component (PC) representing the atherogenic lipoprotein phenotype characteristic of T2D from a major to a secondary component of the total variance. The increase in LDL-C in the CCI group was mainly attributed to larger cholesterol-enriched LDL particles. CIMT showed no change in either the CCI or UC group.

**Conclusion:**

Consumption of a very low carbohydrate diet with nutritional ketosis for 2 years in patients with type 2 diabetes lowered levels of small LDL particles that are commonly increased in diabetic dyslipidemia and are a marker for heightened CVD risk. A corresponding increase in concentrations of larger LDL particles was responsible for higher levels of plasma LDL-C. The lack of increase in total LDL particles, ApoB, and in progression of CIMT, provide supporting evidence that this dietary intervention did not adversely affect risk of CVD.

## Background

Global incidence of diabetes is rising substantially, with the expectation of a 50% increase between 2015 and 2040 [1]. The leading cause of death in patients with diabetes is cardiovascular disease (CVD) [2] and mitigating CVD risk has become a principal focus of current diabetes guidelines [3, 4]. Multiple studies have found that therapeutic carbohydrate restriction significantly improves a number of CVD risk factors [5–7], including elevated triglycerides and small dense LDL, low HDL-C, and markers of non-alcoholic fatty liver disease [8, 9]. These factors contribute to residual risk of CVD following statin treatment for lowering of LDL-cholesterol (LDL-C) [10].

Although LDL-C has been a mainstay for CVD risk prediction and management for decades, it is not characteristically elevated in patients with diabetes. Rather, the most common dyslipidemia in type 2 diabetes (T2D) consists of high triglycerides (TG), low HDL-cholesterol (HDL-C), and a preponderance of small dense LDL particles [11, 12]. This trait which is a central feature of metabolic syndrome, has been designated the atherogenic lipoprotein phenotype (ALP) or atherogenic dyslipidemia [11–14]. Multiple studies have shown beneficial effects of carbohydrate restriction on this phenotype [15–17]. Recently, very low carbohydrate diets that achieve nutritional ketosis have been shown to be of benefit in diabetes management, with effects including improvements in weight, HbA1c, triglycerides and HDL-C [5, 18]. However, such diets often result in increased concentrations of LDL-C [18, 19], which has raised concerns regarding an adverse effect on CVD risk.

While LDL-C is taken to reflect the role of LDL particles in the development of CVD, it has been shown that measurement of LDL particles [20, 21] and ApoB, which is a measure of the number of all atherogenic particles (LDL, IDL, VLDL, lipoprotein (a), chylomicron remnants) [20, 22] can provide superior assessment of CVD risk, most notably when there is discordance between LDL-C and LDL particle concentrations [22]. This discrepancy is commonly due to increased levels of small, dense, cholesterol-depleted particles, as is the case for the dyslipidemia of T2D. There is increasing evidence that levels of small, dense LDL are predictive of CVD incidence independent of LDL-C [23–27], whereas in general levels of large LDL show weak or absent associations with CVD risk [27]. Properties of small LDL that may underlie this risk include increased circulation time due to decreased receptor-mediated uptake, increased vascular wall binding, and increased susceptibility to oxidation and glycation [28]. Assessment of other lipoprotein particle subclasses, including those within VLDL and HDL, has provided the ability to further assess CVD risk [20].

We previously reported that 2 years of treatment with a continuous care intervention (CCI) produced significant improvements in weight, blood glucose, HbA1c, liver function, and inflammatory markers with no adverse effects on kidney markers [29]. Participants in the CCI group had a 0.9% mean absolute reduction in HbA1c and a 10% average weight loss at 2 years [29]. CCI is a personalized carbohydrate restriction (CR) intervention with guidance encouraging nutritional ketosis that is delivered and supported remotely using a telemedicine-approach, via one-to-one health coaching and physician-led treatment. At 1 year, the intervention resulted in substantial improvements in multiple cardiometabolic risk markers including triglycerides, HDL-C, ApoA1, ApoB: ApoA1 ratio, and blood pressure [30, 31]. However, there was an increase in LDL-C that was maintained through 2 years despite sustained improvements in TG and HDL-C [30]. To further characterize changes in LDL and other lipoproteins at 1 and 2 years, we have here utilized the technique of ion mobility (IM) which directly measures concentrations of lipoprotein particle subclasses across the full diameter spectrum from HDL to VLDL. The primary aims were to investigate the effect of the CCI and UC on lipoprotein subfractions and carotid intima-media thickness (CIMT). Secondary aims included: (1) investigating the effect of CCI and UC on T2D atherogenic dyslipidemia using both principal component analyses and assessment of LDL subclass phenotypes and (2) among the CCI participants, comparing the 2-year changes of lipoprotein subclasses and CIMT between individuals in the highest and lowest quartiles of either LDL-C or ApoB responses. Other ancillary aims included assessing potential relationships between adiposity and beta-hydroxybutyrate (BHB) with changes in lipids, lipoproteins and LDL phenotype.

## Materials and methods

### Study design and intervention

The data analyzed for this study are measurements of CVD risk markers obtained at baseline and after 1 and 2 years of follow-up in participants in the clinical trial NCT02519309. This is an open-label, non-randomized controlled trial of the effects of carbohydrate restriction including nutritional ketosis conducted in a cohort of patients with T2D. The study design, comprehensive details of the study intervention, and major exclusion criteria were previously published [29, 31]. Briefly, the trial recruited participants with an established diagnosis of T2D and a body mass index (BMI) > 25 kg/m^2^, who self-selected to receive either the CCI or usual care (UC). All study participants were informed and consented to participate in the study, and the study was approved by the Franciscan Health Lafayette Institutional Review Board. Patients in the CCI group received nutritional advice on carbohydrate restriction to achieve and sustain nutritional ketosis. They were initially advised to consume < 30 g of carbohydrates, approximately 1.5 g protein per kg reference body weight, and fat to satiety each day. Blood beta-hydroxybutyrate (BHB) was used as a marker of carbohydrate restriction, with BHB ≥ 0.5 mM [32] indicating nutritional ketosis. Over time, BHB and dietary intake targets were modified according to patient health needs, goals, and values. The patients had access to a web-interfaced software application (app) that they used to communicate with their remote care team and receive telemedicine-based treatment. The app was used to upload selected biomarkers for monitoring adherence to nutritional intervention and health-related progress including body weight, blood glucose, and BHB. The frequency of reporting glucose and BHB was adjusted to each participant’s preferences and current health needs. Participants with a confirmed history of hypertension additionally received an automatic sphygmomanometer, blood pressure readings were uploaded in the app for assessment by the care team. The reported blood glucose and blood pressure readings were evaluated routinely by the physician who adjusted diabetic and anti-hypertensive prescriptions as needed. Via the app, participants had access to online resources and the opportunity to participate in an online social support community.

Patients who chose UC comprised a reference group that was recruited from the same geographical and healthcare system. They continued with their existing care team without modification and received nutritional and lifestyle advice as recommended by the American Diabetes Association (ADA) between August 2015 and May 2018 [3]. No study-specific modification of treatment or care was made but the participants in the UC arm were required to obtain annual tests for measurement of clinical biomarkers.

### Anthropometric measures

Anthropometric measures were obtained for both CCI and UC participants in the clinic at baseline, 1 year, and 2 years. Body weight and height were measured using a stadiometer and calibrated scale, respectively and the values were used to calculate body mass index (BMI). Manual blood pressure measurements were performed by trained staff. Dual-energy X-ray absorptiometry (DXA; Lunar GE Prodigy, Madison, WI) was utilized to measure total body composition and to estimate central abdominal fat (CAF), as previously described [29], in the CCI group only.

### Lipid analyses

An accredited Clinical Laboratory Improvement Amendment (CLIA) laboratory was used to analyze all the standard blood analytes. For the determination of total cholesterol, HDL-C, TG, ApoA1, and ApoB, an enzymatic, colorimetric method was employed using FDA approved Cobas c501 (Roche Diagnostics; Indianapolis, IN, USA) assays. LDL cholesterol (LDL-C) levels were calculated using the Friedewald equation, except if the TG level exceeded 400 mg/dL, in which case LDL-C was not determined (n = 15, 8, 8 in CCI and n = 9, 10, 6 in UC at baseline, 1 year and 2 years, respectively). Both non-HDL and remnant cholesterol were calculated using simple formulas listed below. ApoB: ApoA1 ratios were computed. Non-HDL cholesterol was calculated as total minus HDL cholesterol and remnant cholesterol was assessed as total cholesterol minus (HDL-cholesterol plus LDL-cholesterol).

$$ {\text{Non - HDL}} = {\text{Total cholesterol}}{-}{\text{HDL - cholesterol}} $$

### Lipoprotein analyses

Particle concentrations of VLDL, IDL, LDL, and HDL subfractions were analyzed in specific particle-size intervals using ion mobility (IM), which uniquely allows for direct particle quantification as a function of particle diameter [21] following a procedure to remove other plasma proteins [24]. The IM instrument utilizes an electrospray to create an aerosol of particles which then pass through a differential mobility analyzer coupled to a particle counter. Particle concentrations (nmol/L) were measured in 11 size intervals (Å): VLDL: large (424.0 to 547.0), medium (335.0 to 424.0), small (296.0 to 335.0); IDL: large (250.0 to 296.0) and small (233.3 to 250.0); LDL: large LDL I (224.6 to 233.3), medium LDL IIa (220.0 to 224.6), and LDL IIb (214.1 to 220.0), small LDL IIIa (208.2 to 214.1) and LDL IIIb (204.9 to 208.2), very small LDL IVa (199.0 to 204.9), LDL IVb (190.0 to 199.0) and LDL IVc (180.0 to 190.0); HDL: large HDL 2b (105.0 to 145.0) and smaller HDL 2a + 3 (76.5 to 105.0). In addition, particles in the size range between LDL and HDL (145.0 to 180.0 Å) were measured (designated mid-zone). Peak LDL diameter was determined as described [22]. Interassay variation was reduced by the inclusion of two in-house controls in each preparatory process and triplicate analysis. Inter- and intra-assay coefficients of variability were < 15% for lipoprotein subclass concentrations and < 0.8% for LDL peak diameter. In addition, LDL subclass phenotypes were determined as described previously [33]: phenotype A (predominance of larger LDL particles with LDL peak diameter > 21.88 nm), phenotype B (predominance of small LDL particles with LDL peak diameter < 21.55 nm), or intermediate phenotype I (with LDL peak diameter between 21.55 and 21.88 nm).

### Carotid intima-media thickness (CIMT) measurement

Ultrasound assessment CIMT was performed in both CCI and UC participants. A high-resolution B mode carotid ultrasound was used (Philips EPIQ5 system; Amsterdam, Netherlands) and the scans were performed by trained and blinded technicians. The participants were placed in a supine position, and both right and left carotid arteries were evaluated with grayscale, spectral, and color Doppler images. The images were taken 1 cm distal to the carotid bulb, below its bifurcation limit. As previously published [30], three imaging planes, anterior, lateral, and posterior, were captured for each participant. These images were then analyzed using the edge detection software (Carotid Analyzer for Research, Medical Imaging Application, Coralville, IA) by a trained and blinded analyst. Any images that were classified “poor” and those with missing planes were removed from all the time points, before the right and left mean CIMT and diameter were calculated from the images. The right and left CIMT average measurements were then used to calculate the overall mean CIMT.

### Statistical analyses

Analyses were performed using IBM SPSS Statistics for Windows, version 26.0 (IBM Corp, Armonk, N.Y., USA). All were first examined for normality and linearity using the skewness and kurtosis cut-offs suggested by Kline’s 2011 guidelines [34]. Four outcomes were positively skewed (i.e., triglycerides, LDL IVa, LDL IVb, and LDL IVc), these variables were normalized by either removal of the top 1% of values or natural log transformations (as specified in the tables’ footnotes). Between-group and between completers versus dropouts' differences in baseline data were analyzed using independent sample t-tests.

All analyses were based on the per-protocol principle including only participants with available data at baseline and 2 years. We used linear mixed-effects models (LMMs) to analyze all the primary endpoints. The models included fixed effects of time, treatment group, and time-by-group interaction to estimate the adjusted means at each time point and to assess the time-effect of the treatments (baseline to 2 years) and between-treatment group differences (CCI versus UC). All models were adjusted for baseline age, sex, BMI, insulin use, statin use, HDL 2 + 3a, and mid-zone. BMI, insulin-use, HDL 2 + 3a and mid-zone were included as co-variates because they differed significantly between CCI and UC groups at baseline. A sensitivity analysis was conducted using data including all participants (262 CCI and 87 UC participants) based on the intent-to-treat principle. The estimation of the missing data in the LMMs was based on the maximum likelihood approach and an unstructured (UN) covariance structure was used to account for within-group correlation over time. The changes in the proportion of participants’ use of lipid-lowering and antihypertensive medications between baseline versus 2 years in both CCI and UC were analyzed using McNemar’s test with continuity correction when appropriate. Logistic generalized estimating equations (GEE) analysis with an unstructured covariance matrix were used to analyze the time-effect of each treatment group (CCI and UC) on the trichotomous categorical variable, LDL phenotype pattern (Pattern A, B and I). Covariates included baseline age, sex, BMI, insulin use, statin use, HDL 2 + 3a, and mid-zone.

Individual differences in the changes of LDL-C and ApoB between baseline and 2 years were assessed using hypo- and hyper-responder categories. For the classification of LDL-C and ApoB hypo- versus hyper-responders, we generated quartiles using the calculated delta LDL-C and delta ApoB from baseline to 2 years. The lowest (greatest decrease) and highest (greatest increase) quartiles were classified as hypo- and hyper-responders, respectively. A-one-way MANOVA was performed to assess the differences in the multivariate lipoprotein profiles at 2 years for the LDL-C and ApoB hypo- versus hyper-responders. Lipoprotein variables that failed the Shapiro–Wilk test of normality were log-transformed before inclusion in the MANOVA to meet the multivariate normality and outliers' assumptions. The 2-year differences in mean CIMT between the LDL-C or ApoB hypo- and hyper-responder groups were assessed using independent T-tests.

Finally, measures of adiposity and BHB were tested as predictors of changes in lipids and lipoproteins. Linear and multiple linear regression analyses were used to assess the relationships between changes in BMI and CAF with lipids and lipoproteins. BHB values that were uploaded in the app by the CCI participants were treated as count data, where the number of days participants reported a BHB value of ≥ 0.5 mM over the past 24 months was modeled using negative binomial regression for association with lipids, lipoproteins, and LDL phenotype shift.

A strict Bonferroni correction was applied to the LMM and MANOVA analyses, where *P* < 0.0015 and *P* < 0.003, respectively indicated statistical significance. For all other exploratory analyses, *P* < 0.05 was used to determine statistical significance.

### Principal component analysis

There was a strong inter-correlation and dependency between the lipoprotein subclasses and lipid variables. To simplify analysis, principal component analysis (PCA) was performed on the 16 lipoproteins and 3 traditional lipids to reduce the variables by generating a new independent combination of the variables that explains the variance of the data. Separate PCAs were performed on baseline and 2-year follow-up data in the CCI and UC treatment groups. Three steps were used: (1) Identification and extraction of major principal components, (2) Rotation of the principal components to identify relevant loading factors, and (3) Interpretation of the principal components and its associated variance. First, we assessed the data for sampling adequacy and its suitability for factor analysis using the Kaiser–Meyer–Olkin (KMO) statistic (cut-off > 0.6) and the Barlett test of sphericity (P < 0.001). Then, we performed PCA on the baseline CCI (n = 223) and UC (n = 70), and 2-year follow-up CCI (n = 140) and UC (n = 46) data, separately. The major principal components represented in each dataset were extracted after assessing the scree plots, and an eigenvalue of 1 was used as a cut-off to select and retain the principal components. We used both varimax and promax rotation methods to identify loading factors for each principal component and a loading value cut-off > 0.40 was used to determine the individual lipoproteins and lipids represented in each component. The individual extracted principal components and their associated variance at baseline and 2-years follow-up were qualitatively assessed. The variance of the individual PCs at baseline and 2-years explains how much of the information in the data is captured by the respective PCs. A PC with the highest variance contribution represents the most information in the data, while a PC with less variance captures less information in the data. Changes in the rank of the PCs were assessed at baseline and 2 years.

## Results

### Participant characteristics

This study enrolled 262 CCI and 87 UC participants, with 194 CCI and 68 UC participants remaining enrolled for 2 years. As previously reported [20], baseline demographic characteristics of the two treatment groups were similar except for the proportion of African Americans. Baseline anthropometric measures, CVD risk markers, and average CIMT were similar between CCI and UC, except for BMI (Additional file 1: Table S1). Baseline levels of lipoprotein subclasses were similar between CCI and UC groups, except for mid-zone and small HDL 2a + 3 which were significantly lower in the CCI group. At baseline, 50% of CCI and 59% of UC participants were on statin treatment (*P* = 0.16). There were no significant differences in baseline characteristics of those who dropped out of the study versus those remaining, except for the baseline proportion of LDL phenotypes in UC (Additional file 1: Table S1).

### Changes in primary laboratory and clinical outcome measures

Within the UC group, no changes over time in lipids, lipoproteins, apoproteins, blood pressure, CIMT and lipid-lowering and anti-hypertensive medications were observed. Among CCI participants at 1 and 2 years, mean LDL-C and HDL-C increased, mean TG and blood pressure decreased, and total cholesterol was unchanged, as previously reported [20] (Additional file 1: Table S2, Figure S1). Lower blood pressures were observed concurrent with reduced use of antihypertensive medication (*P* = 1.0 × 10^–3^), particularly diuretics (*P* = 7.0 × 10^–3^) at 2 years (Additional file 1: Table S3). The use of statin medication was unchanged at 2 years, but the use of other lipid-lowering medications (bile acid sequestrants, fibrates, niacin and omega-3 fatty acid ethyl esters) decreased (*P* = 8.0 × 10^–3^) from a small baseline population of 9.3%.

Among CCI participants, remnant cholesterol decreased at 1 and 2 years (− 22.4% at 2 years, P = 3.1 × 10^–7^), and ApoA1 increased (+ 10.9% at 2 years, *P* = 1.4 × 10^–7^; Additional file 1: Table S2). Non-HDL, ApoB, ApoB: ApoA1 ratio, and CIMT were unchanged (Additional file 1: Table S2). No significant changes in total LDL, total IDL, total VLDL, and total HDL particles were seen in the CCI and UC groups. Among lipoprotein subfractions, VLDL subclasses and IDL I were unchanged at 2 years, while IDL II increased (+ 24.6% at 2 years, *P* = 2.0 × 10^–10^, Table 1, Additional file 1: Figure S1) and was greater than UC (*P* = 5.1 × 10^–8^). Large LDL I increased at one and 2 years (+ 29.1% at 2 years, *P* = 2.4 × 10^–8^) concurrent with increases in LDL peak diameter (+ 2.0% at 2 years, *P* = 1.9 × 10^–10^); both were greater compared to UC (*P* = 2.0 × 10^–6^ and *P* = 1.2 × 10^–4^, respectively). LDL IIa and IIb were unchanged. Small LDL IIIa and IIIb decreased at 1 year, with LDL IIIb maintaining significance at 2 years (− 23.1% at 2 years, *P* = 1.0 × 10^–3^) where it was lower compared to UC (*P* = 1.0 × 10^–3^) (Table 1), while the reduction in LDL IIIa at 2 years was of borderline significance after Bonferroni correction (*P* = 3.0 × 10^–3^). There were non-significant decreases in very small LDL (IVa–c). Particles in the mid-zone were lower at 1 and 2 years (− 6.8% at 2 years, *P* = 7.4 × 10^–7^) and compared to UC (*P* = 1.0 × 10^–3^). No significant differences in HDL subfractions were observed in either CCI or UC groups. An intent-to-treat sensitivity analysis using all available data revealed results consistent with the per-protocol (completers) analysis (Additional file 1: Table S4).

**Table 1:** Adjusted means and changes in lipoproteins over time by treatment group among completers

Variables	Visit	Continuous care intervention (n = 194)		Usual care (n = 68)		Between group effect	
		Mean ± SE	Change from baseline (Mean, CI)	Mean ± SE	Change from baseline (Mean, CI)	Mean difference	95% CI
Total VLDL (nmol/L)	Baseline	138.6 ± 5.7		144.0 ± 8.4		− 5.3	− 25.4 to 14.8
	1 year	128.7 ± 5.8	− 9.9, − 25.8 to 5.9	146.9 ± 8.7	3.0, − 20.3 to 26.2	− 18.2	− 39.0 to 2.5
	2 years	129.3 ± 5.9	− 9.3, − 25.3 to 6.6	144.1 ± 8.7	0.2, − 23.0 to 23.4	− 14.8	− 35.7 to 6.0
VLDL large (nmol/L)	Baseline	23.9 ± 1.0		22.2 ± 1.7		1.7	− 2.3 to 5.7
	1 year	19.1 ± 1.0	− 4.8, − 7.6 to − 2.0*	22.7 ± 1.8	0.5, − 4.3 to 5.2	− 3.6	− 7.7 to 0.6
	2 years	20.3 ± 1.0	− 3.6, − 6.4 to − 0.8	21.9 ± 1.7	− 0.3, − 5.0 to 4.4	− 1.6	− 5.7 to 2.4
VLDL medium (nmol/L)	Baseline	57.3 ± 1.8		55.2 ± 3.1		2.1	− 5.2 to 9.3
	1 year	52.3 ± 1.8	− 5.0, − 10.0 to 0.0	54.6 ± 3.2	− 0.5, − 9.1 to 8.0	− 2.4	− 9.8 to 5.1
	2 years	52.5 ± 1.8	− 4.7, − 9.7 to 0.3	54.7 ± 3.1	− 0.5, − 8.9 to 7.9	− 2.2	− 9.4 to 5.1
VLDL small (nmol/L)	Baseline	59.3 ± 1.5		55.8 ± 2.6		3.4	− 2.6 to 9.5
	1 year	63.2 ± 1.5	3.9, − 0.3 to 8.1	55.0 ± 2.7	− 0.8, − 8.0 to 6.3	8.2	2.0 to 14.4
	2 years	59.2 ± 1.5	− 0.1, − 4.3 to 4.1	54.9 ± 2.6	− 0.9, − 8.0 to 6.1	4.3	− 1.8 to 10.4
Total IDL (nmol/L)	Baseline	256.6 ± 8.0		255.1 ± 11.9		1.6	− 27.0 to 30.1
	1 year	285.1 ± 8.3	28.4, 5.9 to 50.9	256.1 ± 12.3	1.1, − 32.0 to 34.1	28.9	− 0.6 to 58.4
	2 years	288.9 ± 8.4	32.2, 9.6 to 54.9 ^Ï^	261.5 ± 12.3	6.4, − 26.5 to 39.4	27.3	− 2.3 to 57.0
IDL 1 (nmol/L)	Baseline	136.2 ± 3.3		129.2 ± 5.7		7.0	− 6.2 to 20.1
	1 year	139.4 ± 3.3	3.2, − 5.9 to 12.3	128.8 ± 5.9	− 0.4, − 15.9 to 15.0	10.6	− 2.9 to 24.1
	2 years	136.4 ± 3.3	0.2, − 8.9 to 9.2	128.3 ± 5.7	− 0.9, − 16.1 to 14.3	8.0	− 5.1 to 21.1
IDL 2 (nmol/L)	Baseline	122.1 ± 3.8		113.3 ± 6.5		8.8	− 6.3 to 23.8
	1 year	152.1 ± 3.8	30.0, 19.6 to 40.4*	112.0 ± 6.7	− 1.3, − 19.0 to 16.4	40.1*	24.6 to 55.5*
	2 years	156.3 ± 3.8	34.2, 23.9 to 44.6*	114.0 ± 6.5	0.7, − 16.7 to 18.1	42.3*	27.2 to 57.3*
Total LDL (nmol/L)	Baseline	991.8 ± 25.9		1028.7 ± 40.8		− 36.9	− 132.7 to 58.9
	1 year	962.7 ± 26.8	− 29.1, − 101.9 to 43.7	1026.5 ± 43.1	− 2.2, − 117.2 to 112.8	− 63.8	− 164.4 to 36.8
	2 years	1002.0 ± 27.0	10.3, − 62.8 to 83.4	1022.1 ± 42.3	− 6.6, − 120.2 to 107.0	− 20.0	− 119.6 to 79.6
LDL I (nmol/L)	Baseline	163.5 ± 6.0		155.6 ± 10.3		7.9	− 16.0 to 31.8
	1 year	207.7 ± 6.1	44.2, 27.6 to 60.7*	146.1 ± 10.7	− 9.6, − 37.7 to 18.6	61.7*	37.1 to 86.2*
	2 years	211.1 ± 6.0	47.6, 31.1 to 64.0*	152.9 ± 10.3	− 2.8, − 30.4 to 24.9	58.2*	34.3 to 82.1*
LDL IIa (nmol/L)	Baseline	137.8 ± 4.7		135.8 ± 8.0		2.0	− 16.6 to 20.5
	1 year	153.4 ± 4.7	15.6, 2.7 to 28.4	126.5 ± 8.3	− 9.3, − 31.1 to 12.5	26.9	7.8 to 45.9
	2 years	156.1 ± 4.7	18.3, 5.5 to 31.1 ^Ï^	129.2 ± 8.0	− 6.6, − 28.0 to 14.8	26.9^Ï^	8.4 to 45.4^Ï^
LDL IIb (nmol/L)	Baseline	176.0 ± 5.6		170.1 ± 9.5		6.0	− 16.1 to 28.0
	1 year	169.7 ± 5.6	− 6.3, − 21.6 to 9.0	166.0 ± 9.9	− 4.1, − 30.0 to 21.9	3.7	− 19.0 to 26.3
	2 years	174.9 ± 5.6	− 1.1, − 16.3 to 14.2	171.4 ± 9.5	1.3, − 24.3 to 26.9	3.6	− 18.5 to 25.7
LDL IIIa (nmol/L)	Baseline	191.5 ± 7.3		169.8 ± 12.6		21.7	− 7.4 to 50.8
	1 year	157.4 ± 7.4	− 34.0, − 54.2 to − 13.9*	187.2 ± 13.0	17.4, − 16.7 to 51.6	− 29.8	− 59.6 to 0.1
	2 years	161.1 ± 7.3	− 30.3, − 50.4 to − 10.3^Ï^	193.5 ± 12.6	23.8, − 9.9 to 57.4	− 32.4	− 61.5 to − 3.3
LDL IIIb (nmol/L)	Baseline	87.8 ± 4.4		84.7 ± 7.6		3.1	− 14.6 to 20.7
	1 year	66.5 ± 4.5	− 21.2, − 33.5 to − 9.0*	98.0 ± 7.9	13.3, − 7.5 to 34.1	− 31.5*	− 49.6 to − 13.4*
	2 years	67.5 ± 4.4	− 20.3, − 32.5 to − 8.1*	96.5 ± 7.6	11.8, − 8.7 to 32.2	− 29.0*	− 46.6 to − 11.3*
LDL IVa (nmol/L)^a^	Baseline	89.1 ± 3.2		95.0 ± 5.6		− 5.9	− 18.7 to 6.9
	1 year	76.4 ± 3.2	− 12.7, − 21.4 to − 4.0^Ï^	93.1 ± 5.9	− 1.9, − 17.4 to 13.6	− 16.7	− 30.0 to − 3.4
	2 years	76.9 ± 3.2	− 12.2, − 20.9 to − 3.6	89.9 ± 5.6	− 5.1, − 20.2 to 10.1	− 13.1	− 25.9 to − 0.2
LDL IVb (nmol/L)^a^	Baseline	78.3 ± 1.8		82.8 ± 3.2		− 4.5	− 11.8 to 2.8
	1 year	71.6 ± 1.8	− 6.7, − 11.7 to − 1.7	82.7 ± 3.3	− 0.1, − 8.8 to 8.6	− 11.1^Ï^	− 18.6 to − 3.6^Ï^
	2 years	73.2 ± 1.8	− 5.1, − 10.1 to − 0.2	81.5 ± 3.2	− 1.3, − 9.9 to 7.5	− 8.3	− 15.6 to − 1.0
LDL IVc (nmol/L)^a^	Baseline	88.9 ± 1.3		89.6 ± 2.2		− 0.6	− 5.8 to 4.5
	1 year	83.6 ± 1.3	− 5.3, − 8.8 to − 1.7^Ï^	88.6 ± 2.3	− 0.9, − 7.1 to 5.2	− 5.0	− 10.3 to 0.3
	2 years	84.0 ± 1.3	− 5.0, − 8.5 to − 1.4	85.7 ± 2.2	− 3.8, − 9.8 to 2.2	− 1.8	− 6.9 to 3.4
Mid-zone (nmol/L)	Baseline	875.3 ± 8.6		892.2 ± 14.7		− 16.9	− 50.9 to 17.1
	1 year	828.6 ± 8.6	− 46.6, − 70.2 to − 23.1*	890.5 ± 15.2	− 1.7, − 41.8 to 38.3	− 61.8*	− 96.7 to − 26.9*
	2 years	815.4 ± 8.6	− 59.9, − 83.4 to − 36.4*	876.0 ± 14.7	− 16.2, − 55.6 to 23.2	− 60.6*	− 94.6 to − 26.5*
Total HDL	Baseline	22.9 ± 0.3		25.1 ± 0.5		− 2.2	− 3.4 to − 0.9
	1 year	22.8 ± 0.4	− 0.2, − 1.1 to 0.8	24.8 ± 0.5	− 0.3, − 1.7 to 1.1	− 2.0	− 3.3 to − 0.7
	2 years	22.8 ± 0.4	− 0.1, − 1.1 to 0.9	25.3 ± 0.5	0.2, − 1.2 to 1.6	− 2.5	− 3.8 to − 1.2
HDL 2b (µmol/L)	Baseline	6.0 ± 0.1		6.0 ± 0.1		0.0	− 0.3 to 0.2
	1 year	6.3 ± 0.1	0.4, 0.1 to 0.6^Ï^	6.0 ± 0.1	− 0.0, − 0.4 to 0.4	0.3	0.0 to 0.7
	2 years	6.3 ± 0.1	0.3, 0.1 to 0.5	6.1 ± 0.1	0.1, − 0.3 to 0.5	− 0.1	− 0.2 to 0.5
HDL 2a + 3 (µmol/L)	Baseline	17.3 ± 0.2		18.0 ± 0.3		− 0.7	− 1.3 to − 0.1
	1 year	16.8 ± 0.2	− 0.5, − 0.9 to − 0.0	17.5 ± 0.3	− 0.5, − 1.2 to 0.3	− 0.7	− 1.4 to − 0.0
	2 years	17.0 ± 0.2	− 0.3, − 0.8 to 0.1	17.8 ± 0.3	− 0.2, − 0.9 to 0.6	− 0.8	− 1.5 to − 0.2
LDL peak diameters (Å)	Baseline	215.2 ± 0.5		215.0 ± 0.8		0.2	− 1.7 to 2.1
	1 year	219.6 ± 0.5	4.4, 3.1 to 5.7*	215.1 ± 0.8	0.1, − 2.1 to 2.3	4.5*	2.6 to 6.5*
	2 years	219.5 ± 0.5	4.3, 3.0 to 5.6*	215.8 ± 0.8	0.8, − 1.4 to 3.0	3.7*	1.8 to 5.6*

Adjusted means and mean changes were obtained from an analysis using linear mixed-effects model (LMM) controlling for baseline age, sex, race, body mass index, HDL 2 + 3a, mid-zone, insulin use and statin use

*SE* standard error, *CI* 95% confidence interval, *LDL* low density lipoprotein, *HDL* high-density lipoprotein, *IDL* intermediate density lipoprotein, *VLDL* very low-density lipoprotein, *CIMT* carotid intima-media thickness

^a^Variables normalized by removing the top 1% of values. Analyses were conducted excluding the top 1% values, although all cases were included using the maximum likelihood approach

^b^Variables normalized by natural log transformation. Non-transformed and unadjusted means, mean changes, CI and standard errors were provided in the table, but the significance level is calculated from the transformed analysis

^*^P < 0.0015 ensures overall simultaneous significance of P < 0.05 over the 33 variables using Bonferroni correction

^Ï^P < 0.005

### Changes in principal components and LDL subclass phenotypes

Principal component analysis was performed on the baseline and 2-year data separately in the CCI and UC groups. At baseline data, for the CCI group, three major principal components (PC1, PC2, PC3) were extracted accounting for 39.9%, 24.8%, and 12.7% of the total variance (77.4%), respectively. PC1 consisted of contributions from small LDLs (LDL IIIa to LDL IVc), large VLDL, medium VLDL, and TG in the positive direction and HDL-C in the negative direction (Additional file 1: Table S5). Major contributors of PC2 were large and medium LDLs (LDL I to LDL IIb), IDLs, VLDL small, and LDL-C in a positive direction. Finally, contributors of PC3 were HDL subclasses (HDL 2b and HDL 2a + 3), the mid-zone fraction and very small LDL IVc in the positive direction. From the 2-year data in the CCI group, four principal components were extracted explaining 82.7% of the overall variance. The major component explained 39.9% of the total variance and was consistent with PC2 of baseline data (Additional file 1: Table S5). The next two components were both consistent with PC1 of baseline data and were therefore designated PC1a and PC1b, accounting for 22.9% and 13.9% of the variance, respectively. Small and very small LDLs contributed to both PC1a and PC1b with a greater contribution from small LDLs (LDL IIIa and IIIb) in PC1a and very small LDLs (LDL IVa to LDL IVc) in PC1b. PC1a was also represented by all VLDLs (mainly medium and large) and TG in a positive and HDL-C in a negative direction. PC1b was strongly represented by the mid-zone fraction and was also moderately associated with TG, and medium and large VLDLs. The last extracted component explained 6.1% of the variance and corresponded closely to PC3 of the baseline data (Additional file 1: Table S5). PCA on both baseline and follow-up UC data consistently extracted three components which corresponded closely to the PCs extracted from the baseline CCI data, except that HDL-C was not loaded in PC3 in the 2-year follow-up data. The distribution of variance explained by each component was similar at baseline and 2 years.

**Fig. 1:**
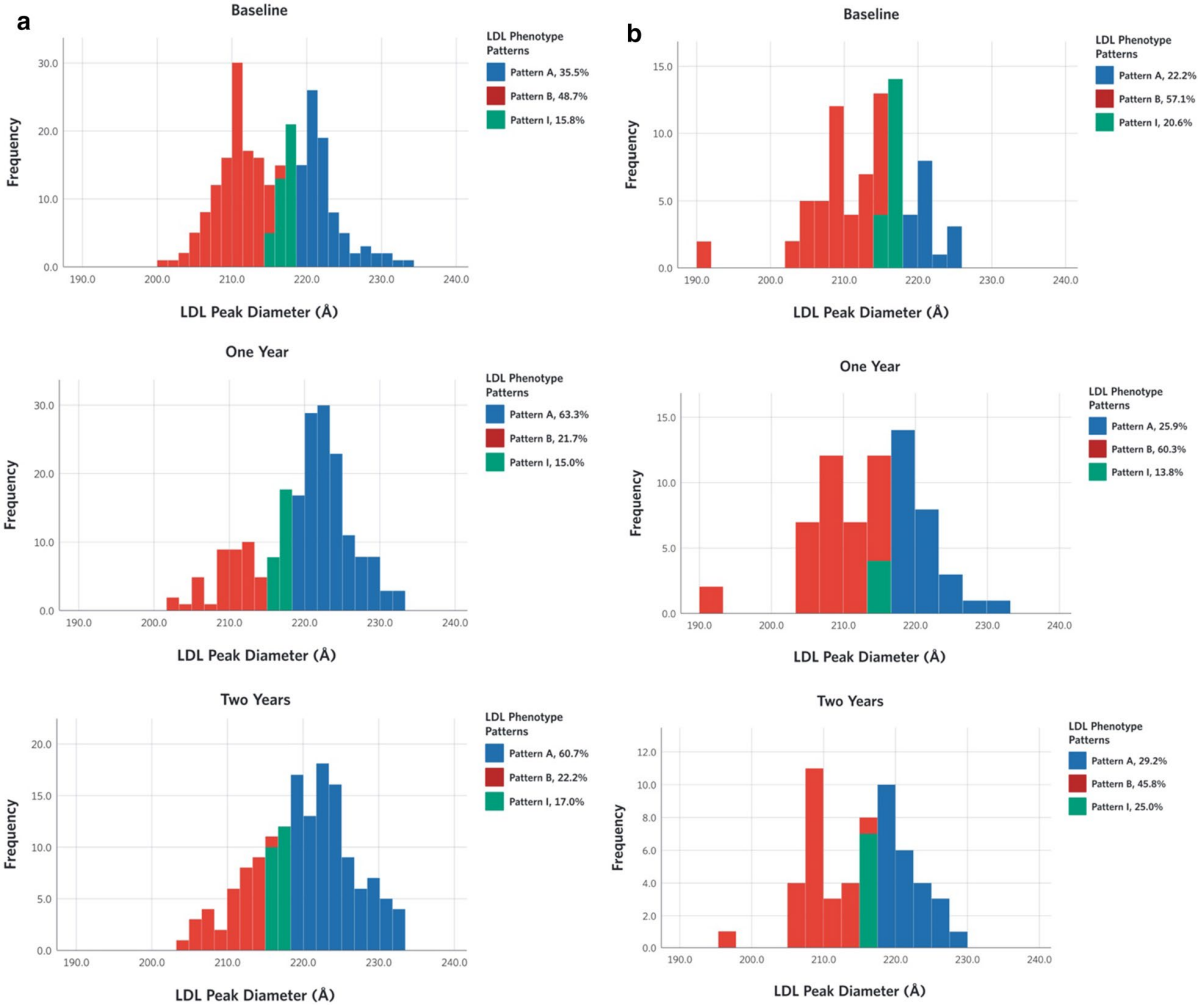
Distribution of LDL phenotype pattern and LDL peak diameters (Å) at baseline, 1 and 2 years. **a** Continuous care intervention, **b** usual care

The distribution of LDL peak diameter and its associated LDL subclass phenotypes among the CCI and UC participants at baseline, 1 and 2 years (Fig. 1) generally indicates a bimodal distribution consistent with the previous categorization of these phenotypes [24]. In the CCI group there was a shift in the proportion of LDL phenotypes from B to A while no changes were seen in the UC group (Fig. 1; Additional file 1: Table S6).

### Changes in lipoprotein subfractions and CIMT in LDL-C and ApoB hypo- and hyper-responders

CCI participants in the quartiles of greatest decrease and increase in LDL-C and in ApoB from baseline to 2 years were categorized into hypo- and hyper-responder groups. One-way MANOVA of the lipoprotein subclasses and LDL peak diameter in LDL-C hypo- versus hyper-responders revealed a significant difference in the overall lipoprotein profile (Pillai’s Trace = 0.66; F = 5.09, *P* = 6.0 × 10^–6^). LDL-C hyper-responders had significantly greater VLDL medium, VLDL small, IDL I, IDL II, LDL I, IIa, and IIb compared to hypo-responders at 2 years (Additional file 1: Table S7). There were no significant differences in the lipoprotein profile between ApoB hypo- and hyper-responders (Pillai’s Trace = 0.37; F = 1.75, *P* = 0.37) Additional file 1: Table S8). No differences in mean CIMT at 2 years between the LDL-C (*P* = 0.49) and ApoB (*P* = 0.43) hypo- versus hyper-responders were observed.

### Relationships between BMI, CAF, and nutritional ketosis with lipids and lipoprotein subclasses and phenotypes

Univariate linear regression analyses revealed significant positive associations of 2 year change in BMI with TG, large VLDL, LDL IIIa, and LDL IIb, and inverse correlations with HDL-C, IDL II, and HDL2b. Changes in CAF were positively associated with TG, large VLDL, mid-zone, LDL IVa, LDL IIIa, LDL IIIb, and LDL IIb, and negatively associated with HDL-C, IDL II, LDL I and HDL2b (Additional file 1: Table S9). Including both BMI and CAF in the multiple linear regression model revealed that only change in BMI was positively associated with TG and LDL IIIa explaining 39.0% and 32.0% of variance, respectively, and change in CAF was inversely associated with HDL-C, IDL II, and HDL 2b, explaining 55.0%, 34.0% and 29.0% of the variance, respectively (Additional file 1: Table S9).

More frequent reporting of nutritional ketosis (BHB ≥ 0.5 mM) over 2 years was associated with greater increases in HDL-C, IDL II, and LDL I, and greater decreases in TG and the mid-zone particle fraction (Additional file 1: Table S10). Additionally, there was a significant association between more frequent reporting of nutritional ketosis with LDL phenotype B to A conversion (Additional file 1: Table S11).

## Discussion

Here, we present analyses of changes in CVD risk markers in patients with type 2 diabetes following a 2-year intervention with a very low carbohydrate diet aimed at achieving nutritional ketosis. We demonstrated that, compared with usual care, the very low carbohydrate diet reduced levels of very small LDL IIIb and increased concentrations of large LDL and the closely related IDL-2 species [35], with no significant change in total LDL particles and ApoB, a measure of all atherogenic lipoproteins. The results demonstrate a sustained improvement of the atherogenic lipoprotein phenotype characteristic of type 2 diabetes that comprises elevated plasma triglyceride and small, dense LDL particles, and reduced HDL-cholesterol [11, 12]. Therapies targeting this dyslipidemia have been reported to mitigate CVD residual risk and decrease CVD events among patients with diabetes [13, 14, 36].

Notably, the significant increase in LDL-C in the CCI group was primarily attributed to an increase in larger cholesterol enriched LDL particles. This is consistent with the finding that, among LDL subfractions, only larger LDL I and medium-sized LDL II, but not smaller particles, were significantly greater at 2 years in those in the upper versus lower quartile of dietary LDL-C response. The increase in larger LDL is likely due, at least in part, to high saturated fat intake, which has been shown to preferentially increase levels of these particles, particularly in the context of reduced carbohydrate intake [15, 16, 37]. Since there is a growing consensus that concentrations of LDL particles and ApoB are superior to LDL-C as predictors of CVD, particularly when there is discordance between LDL-C and the particle measures [22, 38], the present findings, including a lack of increase in total LDL particles and ApoB, provide reassurance that the increase in LDL-C with the dietary intervention does not signify an increase in CVD risk. This inference aligns with the observation in the PURE study, where higher dietary saturated fat consumption was associated with higher LDL-C, but not with higher all-cause or CVD mortality [39]. Furthermore, this supposition is consistent with lack of progression of atherosclerosis in our study as assessed by CIMT. Given the stronger association of small versus large LDL particles with CVD risk [23–26], it remains possible that the reduction of very small LDL and other features of atherogenic dyslipidemia in the CCI group might lead to improvement in atherosclerosis measures with a longer-term intervention. A benefit of the dietary intervention on CVD risk might also be predicted by the observed reductions in remnant cholesterol [28], as well as the increases in HDL-C and the HDL protein ApoAI [40, 41], although recent studies have called into question whether reduced CVD risk can be reliably inferred by an increase in HDL-C [42, 43].

Given the evidence for multiple metabolic relationships among the various lipoprotein classes, we turned to PCA to determine whether the effect of the very low carbohydrate diet could be defined by one or more independent clusters of inter-related changes in lipoprotein subfractions. From the baseline data of both the CCI and UC groups, we identified three independent PCs, all corresponding to PCs previously identified in healthy individuals [26]. The major component in the present study (PC1) is consistent with PC2 in the earlier report, which in turn, closely reflects features of the atherogenic lipoprotein phenotype [26]. Notably, this PC has been associated with increased CVD risk [26] and with chronic kidney disease [44]. Moreover, it has been associated with a 22% increase in the odds of coronary artery calcification (CAC) in individuals with diabetes and metabolic syndrome [45] and with CAC in those with reduced kidney function [44]. With dietary intervention in the CCI group, we found that PC1 shifted from the largest variance contributor at baseline to a secondary variance component. Furthermore, it could then be separated into two sub-components (PC1a and PC1b). Interestingly, small LDL IIIa and IIIb were relatively more strongly loaded onto PC1a, along with triglycerides and medium and large VLDL (positively) and HDL-C (negatively). In contrast, very small LDL IVa to LDL IVb were more strongly loaded onto PC1b, along with moderate loading of triglycerides and medium and large VLDL. These distinctions suggest that the very low carbohydrate intervention may have exposed effects on two independent components of the atherogenic lipoprotein phenotype, involving small and very small LDL particles, respectively. The diet-induced shift in PC1 from the primary to the secondary contributor to the overall variance is consistent with conversion from small LDL phenotype B to phenotype A in a high proportion of the CCI participants. This finding is in line with other studies reporting the reversal of phenotype B to A through down-titration of carbohydrate intake relative to fat intake in healthy individuals [16] and in those with metabolic syndrome treated with an isocaloric low carbohydrate, high fat diet [17].

PC2 in the present study is consistent with the main PC (PC1) previously identified in healthy individuals [26] and is represented by LDL-C as well as large and medium LDL, IDL and small VLDL. Consistent with the increase in LDL-C in the CCI group, we showed that the associated variance in PC2 shifted from a secondary to the major contributor at 2 years. While the implications of this shift for CVD remain uncertain, it is notable that this PC was not found to be associated with CVD risk in healthy individuals [26] or with CAC in those with diabetes or metabolic syndrome [45].

Finally, the minor PC3, which was associated with reduced CVD risk in healthy individuals [26] and represents a spectrum of particles ranging from small HDL2a + 3 and large HDL2b to the smallest LDL species (LDL IVc), was not affected significantly by the dietary intervention. However, there was a trend toward increased HDL2b, which might have contributed to the observed increase in HDL-C and ApoAI.

The ion mobility analysis also identified a novel particle fraction in the size range between LDL and HDL, designated mid-zone, which was significantly reduced in the CCI group. The loading of this fraction onto PC1b suggests that it may represent a feature of this component of the atherogenic lipoprotein phenotype. However, it was also represented in PC3, raising the possibility that it may be heterogeneous, representing contributions from both LDL and HDL, and perhaps other particles in this size range. Further studies will be required to characterize this fraction and determine its metabolic significance and possible relation to CVD risk.

The findings from this study raise the question as to the extent to which reduced body weight and central adiposity may have contributed to the lipoprotein changes induced by the very low carbohydrate diet [15, 46]. Although the study design makes it difficult to disentangle these influences, we found that weight loss, abdominal fat reduction, and ketosis were differentially associated with specific lipoprotein particle changes. Both reduction in BMI and more frequent ketosis were correlated with improvement in TG, and reduced BMI was associated with lower levels of small LDLs. On the other hand, ketosis was related to increased large LDL I and conversion of LDL phenotype B to A, and, along with reduced central adiposity, to increased IDL 2 (closely related to large LDL [35]) and HDL-C. We speculate that carbohydrate restriction in conjunction with weight loss either through additive or synergistic actions may reduce the availability of the hepatic triglyceride pool for production of VLDL precursors of small LDLs [15]. On the other hand, more frequent ketosis may reflect greater carbohydrate restriction and higher intake of fat, including saturated fat which, as noted above, preferentially increases level of larger LDL particles in conjunction with reduced carbohydrate intake [15, 16, 37]. One or both of these dietary effects may enhance the conversion of LDL phenotype B to A [16, 47]. Interestingly, a study performed in obese patients who underwent laparoscopic adjustable gastric banding failed to show significant changes in LDL levels and LDL subfractions despite a substantial weight loss of 13.4% at 13 months [48]. Together, these observations, along with earlier studies [15, 17] suggest that carbohydrate restriction and nutritional ketosis may contribute significantly to the observed lipoprotein changes independent of changes in adiposity.

A strength of this study is its 2-year duration, the longest to evaluate lipoprotein changes in response to a very low carbohydrate diet including nutritional ketosis. While free-living ad libitum food consumption among participants who self-selected their intervention enhances the generalizability of the study by mimicking patient choice in lifestyle intervention for diabetes treatment. Within the CCI group, long term tracking of blood BHB as a marker of carbohydrate restriction provided the opportunity to explore the relationship between frequency of reported nutritional ketosis status and shift from LDL subclass phenotype B to A.

A limitation of this study is the lack of randomization and lack of tight control over the food consumed by the CCI and UC groups. In addition, the fact that the study participants were mostly Caucasian limits the generalizability of the study to other races and ethnic groups. Finally, the lack of changes in CIMT in the two groups could be due to insufficient duration of the study or to variation in image acquisition and interpretation among the individuals performing this technique. Furthermore, the CIMT analysis did not include carotid plaque assessment.

## Conclusion

In conclusion, these results demonstrate that in patients with type 2 diabetes, consumption of a very low carbohydrate diet with nutritional ketosis for 2 years was associated with sustained improvement in the atherogenic lipid and lipoprotein profile that is characteristic of this condition. This finding was reinforced by the use of an unbiased principal component analysis that identified this profile as one of three independent clusters of lipoprotein fractions, another of which accounted for the diet-induced increase in LDL-C. While the implications of these effects for CVD outcomes will require future long-term studies, both the lack of increase in total LDL particle number and carotid intima-media thickness point to the cardiovascular safety of a very low carbohydrate diet in the context of a substantial benefit for management of type 2 diabetes [18].

## Supplementary information

**Additional file 1: Figure S1. **Change in lipids and lipoprotein subclasses in CCI at baseline, one and two years. **Table S1. **Baseline characteristics. **Table S2. **Adjusted means and changes in lipids, blood pressure and CIMT over time by treatment group among completers. **Table S3. **Lipid lowering and anti-hypertensive medication use over time among completers. **Table S4.** Adjusted means and changes in lipids, lipoproteins, apoproteins, blood pressure, and CIMT over time by treatment group (intent-to-treat analysis). **Table S5. **Principal Components and Respective Loading of Each Lipoprotein/Lipid from Baseline and Two-year Follow-up Data. **Table S6.** Estimated mean proportions and standard errors in the change of LDL phenotype patterns from baseline to two years. **Table S7.** Multivariate analysis of variance (MANOVA) of lipoprotein subclasses in LDL-C hypo- versus hyper-responders. **Table S8.** Multivariate analysis of variance (MANOVA) of lipoprotein subclasses in ApoB hypo- versus hyper-responders. **Table S9. **Relationships between change in BMI and central abdominal fat with lipids and lipoproteins. **Table S10. **Association between frequency of participants reporting BHB ≥ 0.5mM with change in lipids and lipoproteins from baseline to 2 years. **Table S11**. LDL phenotype conversions and their associations with frequency of participants reporting BHB ≥ 0.5mM from baseline to 2 years.

## Data Availability

The datasets used and/or analysed during the current study are available from the corresponding author on reasonable request.

## Abbreviations

CCI: Continuous care intervention
UC: Usual care
T2D: Type 2 diabetes
CVD: Cardiovascular disease
BMI: Body mass index
BHB: Beta-hydroxybutyrate
HDL-C: High density lipoprotein cholesterol
LDL-C: Low density lipoprotein cholesterol
TG: Triglycerides
IDL: Intermediate density lipoprotein
VLDL: Very large density lipoprotein
ApoB: Apolipoprotein B
ApoA1: Apolipoprotein A1
ALP: Atherogenic lipoprotein phenotype
CR: Carbohydrate restriction
CAF: Central abdominal fat
IM: Ion mobility
CIMT: Carotid-artery intima-media thickness
DXA: Dual-energy X-ray absorptiometry
LMM: Linear mixed-effect model
PCA: Principal component analysis
PC: Principal component
MANOVA: Multivariate analysis of variance
ADA: American Diabetes Association
