# Correction to: Impact of a 2‑year trial of nutritional ketosis on indices of cardiovascular disease risk in patients with type 2 diabetes

**DOI:** 10.1186/s12933-021-01214-9

**Published:** 2021-02-05

**Authors:** Shaminie J. Athinarayanan, Sarah J. Hallberg, Amy L. McKenzie, Katharina Lechner, Sarah King, James P. McCarter, Jeff S. Volek, Stephen D. Phinney, Ronald M. Krauss

**Affiliations:** 1 Virta Health, 501 Folsom Street, San Francisco, CA 94105 USA; 2 Indiana University Health Arnett, Lafayette, IN USA; 3 School of Medicine, Indiana University, Indianapolis, IN USA; 4 Department of Cardiology, German Heart Centre Munich, Technical University Munich, Munich, Germany; 5 DZHK (German Centre for Cardiovascular Research), Munich Heart Alliance, Partner Site Munich, Munich, Germany; 6 School of Medicine, University of California, San Francisco, CA 94143 USA; 7 Abbott Diabetes Care, Alameda, CA 94502 USA; 8 Department of Genetics, Washington University School of Medicine, St. Louis, MO USA; 9 Department of Human Sciences, The Ohio State University, Columbus, OH USA

## Correction to: Cardiovasc Diabetol (2020) 19:208 10.1186/s12933-020-01178-2

Following publication of the original article [1], the author noticed an error in the last sentence of "Lipid analyses" under Methods section. The last sentence should read, “ApoB: ApoA1 ratios were computed. Non-HDL cholesterol was calculated as total minus HDL cholesterol and remnant cholesterol was assessed as total cholesterol minus (HDL-cholesterol plus LDL-cholesterol)”.

The original article has been corrected.
