# Supplementary material for: Impact of a 2-year trial of nutritional ketosis on indices of cardiovascular disease risk in patients with type 2 diabetes
Source: Cardiovasc Diabetol. 2020 Dec 8;19:208. doi: 10.1186/s12933-020-01178-2 (PMC7724865; doi:10.1186/s12933-020-01178-2)
Supplement: Supplementary file 1 — Additional file 1: Figure S1. Change in lipids and lipoprotein subclasses in CCI at baseline, one and two years. Table S1. Baseline characteristics. Table S2. Adjusted means and changes in lipids, blood pressure and CIMT over time by treatment group among completers. Table S3. Lipid lowering and anti-hypertensive medication use over time among completers. Table S4. Adjusted means and changes in lipids, lipoproteins, apoproteins, blood pressure, and CIMT over time by treatment group (intent-to-treat analysis). Table S5. Principal Components and Respective Loading of Each Lipoprotein/Lipid from Baseline and Two-year Follow-up Data. Table S6. Estimated mean proportions and standard errors in the change of LDL phenotype patterns from baseline to two years. Table S7. Multivariate analysis of variance (MANOVA) of lipoprotein subclasses in LDL-C hypo- versus hyper-responders. Table S8. Multivariate analysis of variance (MANOVA) of lipoprotein subclasses in ApoB hypo- versus hyper-responders. Table S9. Relationships between change in BMI and central abdominal fat with lipids and lipoproteins. Table S10. Association between frequency of participants reporting BHB ≥ 0.5mM with change in lipids and lipoproteins from baseline to 2 years. Table S11. LDL phenotype conversions and their associations with frequency of participants reporting BHB ≥ 0.5mM from baseline to 2 years. [file 12933_2020_1178_MOESM1_ESM.docx]

**Impact of a two-year trial of nutritional ketosis on indices of cardiovascular disease risk in patients with type 2 diabetes**

**Running title:** Nutritional ketosis and CVD risk indices in T2D

*Shaminie J. Athinarayanan PhD^1*^, Sarah J. Hallberg DO^1,2,3*^, Amy L. McKenzie PhD^1^, Katharina Lechner MD ^4,5^, Sarah M. King PhD ^6^ James P. McCarter MD, PhD ^7,8^ Jeff S. Volek PhD^1,9^, Stephen D. Phinney MD, PhD^1^, Ronald M. Krauss MD ^6^*

*SJA and SJH contributed equally to the study

^1^ Virta Health, 501 Folsom Street, San Francisco, CA 94105, USA

^2^ Indiana University Health Arnett, Lafayette, IN, USA

^3^ Indiana University, School of Medicine, Indianapolis, IN, USA

^4^ Department of Cardiology, German Heart Centre Munich, Technical University Munich, Munich, Germany

^5^ DZHK (German Centre for Cardiovascular Research), Partner site Munich, Munich Heart Alliance, Munich, Germany

^6^ School of Medicine, University of California, San Francisco, CA 94143, USA

^7^ Abbott Diabetes Care, Alameda, CA 94502, USA

^8^ Department of Genetics, Washington University School of Medicine, St. Louis, MO, USA

^9^ Department of Human Sciences, The Ohio State University, Columbus, OH, USA

Additional Tables: 11

Additional Figures: 1

**Figure S1.** Change in lipids and lipoprotein subclasses in CCI at baseline, one and two years

1. LDL cholesterol,
2. HDL cholesterol
3. Triglycerides
4. Small/very small LDL subclasses
5. Large/medium LDL subclasses
6. Large/medium HDL subclasses
7. IDL subclasses
8. VLDL subclasses

******* p<0.0015; ****** p<0.005

**Figure S1.**

1. **B.**


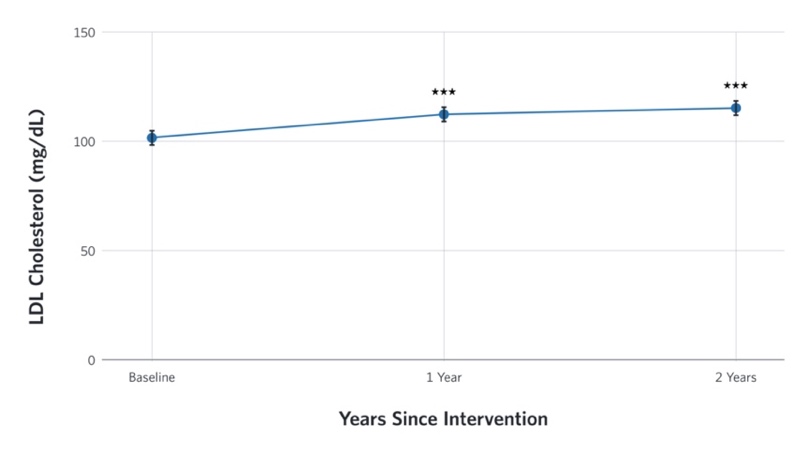

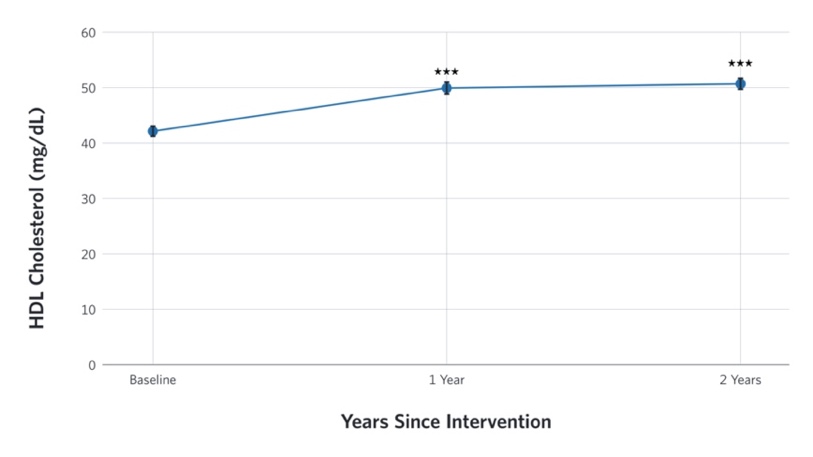


**C. D.**


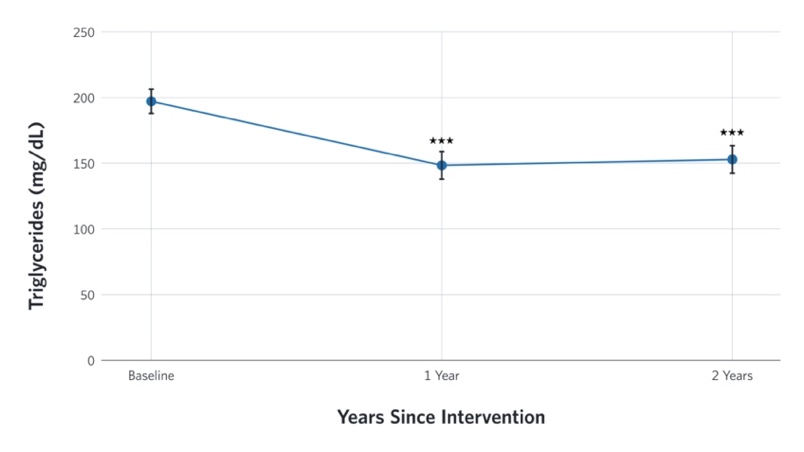

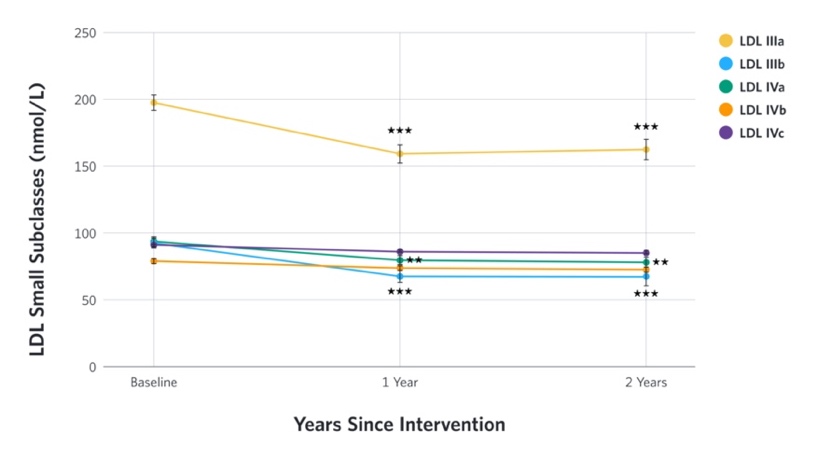


**E. F.**


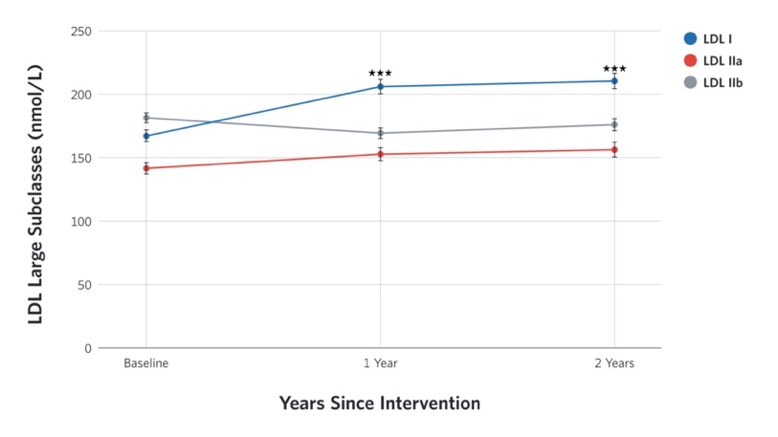

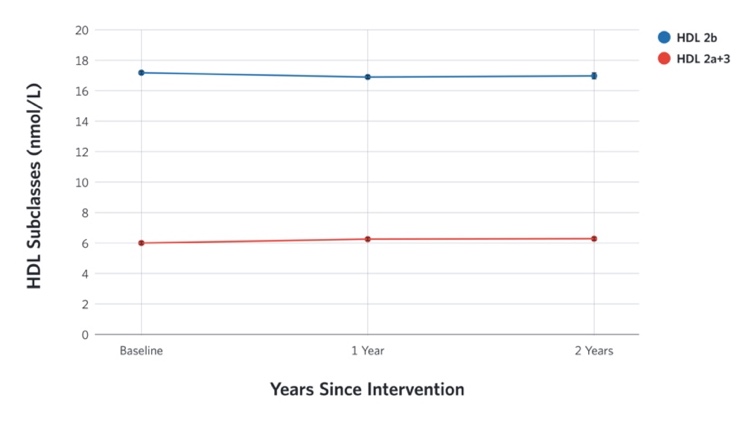


**G. H.**


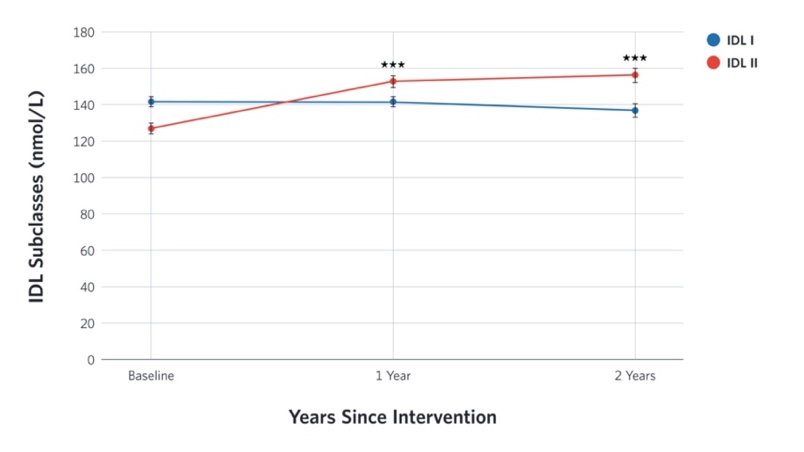

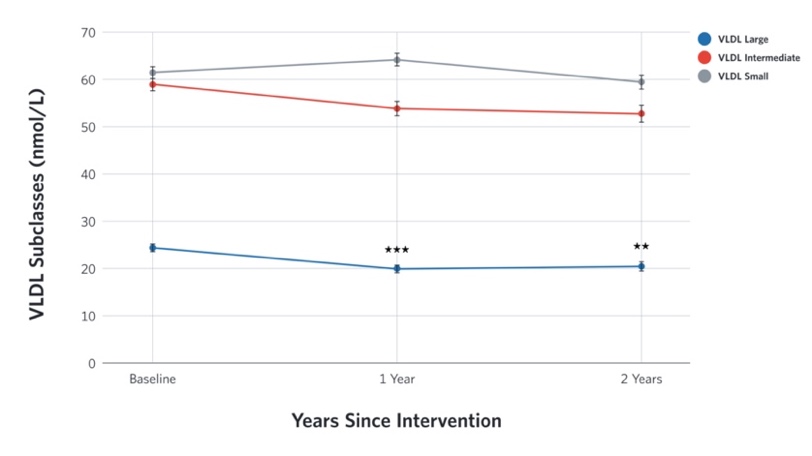


**Table S1.** Baseline characteristics

|  | **All** |  | **Completers with data** |  | **Dropout or missing data** |  |
| --- | --- | --- | --- | --- | --- | --- |
|  | **N** | **Mean (SD) or ±SE** | **N** | **Mean (SD) or ±SE** | **N** | **Mean (SD) or ±SE** |
| Age (years)  CCI  Usual Care  CCI vs. usual care | 262  87 | 53.8(8.4)  52.3(9.5)  1.4±1.1 | 194  68 | 54.4(8.2)  51.4(9.4)  3.0±1.2 | 68  19 | 51.9(8.7) 55.6(9.5)  -3.6±2.4 |
| African American (%)  CCI  Usual Care  CCI vs. usual care | 262  87 | 6.9±1.6  0.0±0.0  6.9±1.6* | 194  68 | 6.2±1.7  0.0±0.0  6.2±1.7* | 68  19 | 8.8±3.5  0.0±0.0  8.8±3.5 |
| Body mass index (kg/m^2^)  CCI  Usual Care  CCI vs. usual care | 257  83 | 40.42(8.81)  36.72(7.26)  3.70±1.07* | 190  64 | 40.41(8.42)  36.90(7.41)  3.51±1.18 | 67  19 | 40.46(9.90)  36.11(6.89)  4.34±2.43 |
| Female (%)  CCI  Usual Care  CCI vs. usual care | 262  87 | 66.79±2.92  58.62±5.31  8.17±6.06 | 194  68 | 65.98±3.41  60.29±5.98  5.69±6.76 | 68  19 | 69.12±5.64  52.63±11.77  6.49±12.35 |
| Years since type 2 diabetes diagnosis  CCI  Usual Care  CCI vs. usual care | 261  71 | 8.44(7.22)  7.85(7.32)  0.59±0.97 | 193  63 | 8.15(7.02)  7.90(7.41)  0.25±1.03 | 68  8 | 9.25(7.75)  7.38(7.05)  1.88±2.87 |
| Systolic blood pressure (mmHg)  CCI  Usual Care  CCI vs. usual care | 260  79 | 131.9(14.1)  129.8(13.6)  2.1±1.8 | 192  61 | 132.2(14.2)  129.0(13.6)  3.3±2.1 | 68  18 | 131.1(13.8)  132.7(13.5)  -1.6±3.6 |
| Diastolic blood pressure (mmHg)  CCI  Usual Care  CCI vs. usual care | 260  79 | 82.1(8.3)  82.0(8.9)  0.1±1.1 | 192  61 | 81.7(8.0)  82.1(8.8)  -0.4±1.2 | 68  18 | 83.4(8.9)  81.8(9.6)  1.6±2.4 |
| Total cholesterol (mg/dL)  CCI  Usual Care  CCI vs. usual care | 247  79 | 183.6(41.2)  183.8(45.8)  -0.2±5.5 | 184  62 | 181.9(40.3)  186.5(49.3)  -4.6±6.3 | 63  17 | 188.7(43.6)  174.0(28.7)  14.7±11.2 |
| LDL-C(mg/dL)  CCI  Usual Care  CCI vs. usual care | 232  70 | 102.5(32.9)  101.5(36.2)  1.0±4.6 | 173  56 | 101.1(33.0)  103.8(38.3)  -2.7±5.3 | 59  14 | 106.6(32.6)  92.3(24.8)  14.3±9.3 |
| HDL-C(mg/dL)  CCI  Usual Care  CCI vs. usual care | 247  79 | 42.2(13.4)  37.6(11.2)  4.6±1.7 | 184  62 | 42.5(13.7)  38.3(11.5)  4.2±1.9 | 63  17 | 41.3(12.7)  35.2(10.1)  6.1±3.3 |
| Triglycerides (mg/dL)  CCI  Usual Care  CCI vs. usual care | 247  79 | 197.2(143.4)  282.9(401.2)  -85.7±46.1 | 184  62 | 200.7(153.5)  283.7(443.6)  -83.0±57.5 | 63  17 | 187.1(109.0)  280.0(185.0)  -92.9±46.9 |
| non-HDL (mg/dL)  CCI  Usual Care  CCI vs. usual care | 247  79 | 141.4(41.1)  146.2(46.5)  -4.7±5.5 | 184  62 | 139.4(39.8)  148.2(50.7)  -8.8±7.1 | 63  17 | 147.4(44.4)  138.8(26.1)  8.6±11.3 |
| Remnant cholesterol (mg/dL)  CCI  Usual Care  CCI vs. usual care | 232  70 | 34.2(15.1)  37.4(16.6)  -3.2±2.1 | 173  56 | 34.5(15.6)  36.3(16.6)  -1.8±2.4 | 59  14 | 33.1(13.8)  41.9(16.5)  -8.8±4.3 |
| VLDL Large (nmol/L)  CCI  Usual Care  CCI vs. usual care | 240  80 | 23.3(13.8)  27.6(16.0)  -4.4±1.9 | 179  62 | 22.7(13.8)  26.4(16.1)  -3.7±2.1 | 61  18 | 24.8(13.6)  31.7(15.5)  -6.9±3.8 |
| VLDL Intermediate (nmol/L)  CCI  Usual Care  CCI vs. usual care | 240  80 | 56.6(24.3)  64.7(28.2)  -8.1±3.3 | 179  62 | 55.3(24.5)  63.4(29.7)  -8.0±3.8 | 61  18 | 60.2(23.7)  69.1(22.5)  -8.9±6.3 |
| VLDL Small (nmol/L)  CCI  Usual Care  CCI vs. usual care | 240  80 | 59.7(18.9)  62.5(21.9)  -2.9±2.5 | 179  62 | 58.2(18.7)  62.5(23.7)  -4.3±3.0 | 61  18 | 64.1(19.2)  62.7(14.7)  1.4±4.2 |
| IDL 1 (nmol/L)  CCI  Usual Care  CCI vs. usual care | 240  80 | 137.7(42.1)  143.4(44.2)  -5.7±5.5 | 179  62 | 134.3(40.9)  143.6(48.2)  -9.3±6.3 | 61  18 | 147.9(44.1)  142.8(27.0)  5.1±11.0 |
| IDL 2 (nmol/L)  CCI  Usual Care  CCI vs. usual care | 240  80 | 125.2(41.3)  119.5(38.5)  5.7±5.2 | 179  62 | 123.1(41.6)  122.9(42.1)  0.2±6.2 | 61  18 | 131.5(40.1)  107.8(19.0)  23.7±6.8* |
| LDL I (nmol/L)  CCI  Usual Care  CCI vs. usual care | 240  80 | 166.2(68.0)  156.9(69.6)  9.3±8.8 | 179  62 | 164.4(69.2)  163.6(75.2)  0.9±10.4 | 61  18 | 171.4(64.7)  133.8(39.1)  37.6±16.1 |
| LDL IIa (nmol/L)  CCI  Usual Care  CCI vs. usual care | 240  80 | 140.8(59.8)  139.3(63.8)  1.5±7.9 | 179  62 | 138.0(59.0)  142.7(67.8)  -4.7±9.0 | 61  18 | 149.1(61.9)  127.6(47.4)  21.5±15.8 |
| LDL IIb (nmol/L)  CCI  Usual Care  CCI vs. usual care | 240  80 | 179.6(70.2)  190.5(82.8)  -10.9±-9.5 | 179  62 | 175.4(67.4)  188.9(86.3)  -13.4±10.7 | 61  18 | 191.8(77.0)  196.3(71.3)  -4.5±20.3 |
| LDL IIIa (nmol/L)  CCI  Usual Care  CCI vs. usual care | 240  80 | 192.0(95.1)  207.6(92.0)  -15.7±12.2 | 179  62 | 189.7(91.4)  202.2(96.1)  -12.5±13.6 | 61  18 | 198.6(106.0)  226.4(75.5)  -27.9±26.8 |
| LDL IIIb (nmol/L)  CCI  Usual Care  CCI vs. usual care | 240  80 | 87.0(57.2)  100.1(58.1)  -13.1±7.4 | 179  62 | 87.3(54.7)  98.7(62.1)  -11.3±8.4 | 61  18 | 86.1(64.5)  105.1(42.4)  -19.0±16.2 |
| LDL IVa (nmol/L)  CCI  Usual Care  CCI vs. usual care | 240  80 | 91.6(51.5)  114.9(70.8)  -23.3±8.6 | 179  62 | 92.3(51.4)  114.5(76.8)  -22.2±10.5 | 61  18 | 89.5(52.4)  116.2(46.6)  -26.7±13.7 |
| LDL IVb (nmol/L)  CCI  Usual Care  CCI vs. usual care | 240  80 | 79.6(28.6)  96.9(45.1)  -17.3±5.4 | 179  62 | 79.5(29.3)  94.1(44.2)  -14.5±6.0 | 61  18 | 79.7(26.6)  106.7(48.3)  -27.0±8.8 |
| LDL IVc (nmol/L)  CCI  Usual Care  CCI vs. usual care | 240  80 | 89.5(21.7)  101.7(35.8)  -12.2±4.2 | 179  62 | 88.9(22.3)  99.3(33.3)  -10.4±3.8 | 61  18 | 91.2(19.8)  109.8(43.3)  -18.6±10.5 |
| Mid Zone (nmol/L)  CCI  Usual Care  CCI vs. usual care | 240  80 | 878.1(161.5)  948.8(179.7)  -70.7±21.5* | 179 62 | 869.7(165.4) 930.6(180.9)  -60.9±25.0 | 61  18 | 902.5(148.0)  1011.3(165.3)  -108.8(40.8) |
| HDL 2b (µmol/L)  CCI  Usual Care  CCI vs. usual care | 240  80 | 5.9(1.2)  6.3(0.9)  -0.3±0.1 | 179  62 | 5.9(1.2)  6.3(1.0)  -0.4±0.2 | 61  18 | 6.1(1.3)  6.3(0.9)  -0.1±0.3 |
| HDL 2a+3 (µmol/L)  CCI  Usual Care  CCI vs. usual care | 240  80 | 16.8(2.6)  19.3(2.8)  -2.5±0.3* | 179  62 | 16.8(2.6)  19.3(2.8)  -2.5±0.4* | 61  18 | 16.9(2.5)  19.4(2.5)  -2.5±0.7* |
| LDL peak diameter (Å)  CCI  Usual Care  CCI vs. usual care | 240  80 | 215.7(6.2)  213.3(6.5)  2.4±0.8 | 179  62 | 215.6(6.3)  213.9(6.4)  1.7±0.9 | 61  18 | 215.9(6.0)  211.3(6.4)  -4.6±1.6 |
| Total ApoB  CCI  Usual Care  CCI vs. usual care | 248  79 | 105.1(28.8)  106.9(28.3)  -1.9±3.7 | 185  62 | 104.2(28.7)  108.3(31.0)  -4.2±4.3 | 63  17 | 107.7(29.4)  101.8(13.7)  5.9±7.4 |
| Total ApoA1  CCI  Usual Care  CCI vs. usual care | 248  79 | 145.7(27.6)  148.7(21.5)  -3.0±3.4 | 185  62 | 146.0(28.6)  149.5(22.0)  -3.5±4.0 | 63  17 | 145.0(24.8)  145.9(19.9)  -0.9±6.5 |
| ApoB:Apo A1 Ratio  CCI  Usual Care  CCI vs. usual care | 248  79 | 0.7(0.2)  0.7(0.2)  0.0±0.0 | 185  62 | 0.7(0.2)  0.7(0.3)  -0.0±0.0 | 63  17 | 0.8(0.2)  0.7(0.1)  0.1±0.1 |
| LDL Phenotype Pattern A (%)  CCI  Usual Care  CCI vs. usual care | 201  62 | 41.8±3.5  25.8±5.6  16.0±6.6 | 151  50 | 43.0±4.0  32.0±6.7  11.0±7.8 | 50  12 | 38.0±6.9  0.0±0.0  38.0±6.9 * |
| LDL Phenotype Pattern B (%)  CCI  Usual Care  CCI vs. usual care | 201  62 | 58.2±3.5  74.2±5.6  -16.0±6.6 | 151  50 | 57.0±4.0  68.0±6.7  11.0±7.8 | 61  18 | 62.0±6.9  100.0±0.0  38.0±6.9 * |
| Statin (%)  CCI  Usual Care  CCI vs. usual care | 262  87 | 50.0±5.3  59.0±3.1  -8.6±6.1 | 194  68 | 53.0±3.6  54.0±6.1  -1.8±7.1 | 68  19 | 43.0±6.0  74.0±10.4  -31.0±12.7 |
| Any antihypertensive medication (%)  CCI  Usual Care  CCI vs. usual care | 262  87 | 77.5±2.6  65.5±5.1  12.0±5.4 | 194  68 | 76.8±3.0  63.2±5.9  13.6±6.6 | 68  19 | 79.4±4.9  73.7±10.4  5.7±10.8 |
| ACE and ARB (%)  CCI  Usual Care  CCI vs. usual care | 262  87 | 65.0±3.0  55.0±5.4  9.7±6.0 | 194  68 | 62.0±3.5  51.0±6.1  10.4±6.9 | 68  19 | 74.0±5.4  68.0±11.0  -5.1±11.7 |
| Diuretics (%)  CCI  Usual Care  CCI vs. usual care | 262  87 | 41.0±3.0  29.0±4.9  12.1±6.0 | 194  68 | 41.0±3.5  24.0±5.2  17.2±6.3 | 68  19 | 41.0±6.0  47.0±11.8  -6.2±13.0 |
| Other Lipid Lowering Medication (%)  CCI  Usual Care  CCI vs. usual care | 262  87 | 10.3±1.9  19.5±4.3  -9.2±4.7 | 194  68 | 9.3±2.1  17.6±4.7  -8.4±4.5 | 68  19 | 13.2±4.1  26.3±10.4  -13.1±11.1 |

*Note.* Abbreviations: SD, standard deviation; SE, standard error; CCI, continuous care intervention; UC, usual care; LDL, low-density lipoprotein; HDL, high-density lipoprotein; IDL, intermediate density lipoprotein; VLDL, very low density lipoprotein; ACE, angiotensin converting enzyme inhibitors; ARB, angiotensin-receptor blockers.

For continuous variables, mean and standard deviations were provided and for categorical variables, percent and standard errors were provided

Differences between means or percentages were given with + 1 standard error of the difference

*A significance level of P<0.0015 ensures overall simultaneous significance of P < 0.05 over the 33 variables using Bonferroni correction.

**Table S2.** Adjusted means and changes in lipids, blood pressure and CIMT over time by treatment group among completers

| Variables | Visit | Continuous Care Intervention (n=194) | | Usual Care (n=68) | | Between Group Effect | |
| --- | --- | --- | --- | --- | --- | --- | --- |
|  |  | **Mean ± SE** | **Change from baseline (Mean, CI)** | **Mean ± SE** | **Change from baseline (Mean, CI)** | **Mean**  **Difference** | **95% CI** |
| Lipids, blood pressure, and CIMT | | | | | | | |
| Total Cholesterol (mg/dL) | **Baseline** | 184.0±3.4 |  | 174.2±6.2 |  | 9.8 | -4.3 to 24.0 |
|  | **1 year** | 194.8±3.5 | 10.8, 1.3 to 20.2 | 168.5±6.7 | -5.8, -23.0 to 11,5 | 26.4 * | 11.3 to 41.4 * |
|  | **2 years** | 195.6±3.4 | 11.6, 2.3 to 20.8 | 171.9±6.2 | -2.3, -18.9 to 14.2 | 23.7 * | 9.6 to 37.8 * |
| LDL-C(mg/dL) | **Baseline** | 102.7 ±2.8 |  | 99.2 ±5.1 |  | 3.5 | -8.0 to 15.0 |
|  | **1 year** | 114.0 ±2.8 | 11.3, 3.6 to 19.0 * | 87.6 ±5.5 | -11.6, -25.8 to 2.6 | 26.4 * | 14.0 to 38.8 * |
|  | **2 years** | 115.0 ±2.8 | 12.3,4.7 to 19.9 * | 89.6±5.1 | -9.6, -23.1 to 4.0 | 25.4 * | 13.8 to 36.9 * |
| HDL-C(mg/dL) | **Baseline** | 43.3±1.1 |  | 38.2±1.9 |  | 5.2 | 0.8 to 9.6 |
|  | **1 year** | 51.7±1.1 | 8.4, 5.5 to 11.3 * | 35.1±2.1 | -3.1, -8.4 to 2.3 | 16.6 * | 11.9 to 21.3 * |
|  | **2 years** | 51.2±1.1 | 7.9, 5.0 to 10.8 * | 40.9±1.9 | 2.8, -2.4 to 7.9 | 10.3* | 5.9 to 14.7 |
| Triglycerides (mg/dL)^b^ | **Baseline** | 196.0±12.1 |  | 226.5±45.4 |  | -30.5 | -96.1 to 35.1 |
|  | **1 year** | 137.2±9.2 | -58.8, -36.2 to -82.3 * | 239.5±34.2 | 13.0, -95.3 to 96.6 | -102.3 * | -173.5 to –31.1 * |
|  | **2 years** | 154.8±10.8 | -41.2, -14.7 to -67.7 * | 210.8±19.9 | -15.7, -99.3 to 41.6 | -56.0 | -99.8 to –12.3 |
| Systolic Blood Pressure (mmHg) | **Baseline** | 131.9±1.1 |  | 130.1±2.0 |  | 1.7 | -2.9 to 6.4 |
|  | **1 year** | 125.6±1.2 | -6.2, -9.3 to –3.1 * | 128.8±2.0 | -1.3, -6.7 to 4.0 | -3.2 | -7.9 to 1.6 |
|  | **2 years** | 125.8±1.1 | -6.1, -9.1 to –3.1 * | 129.9±2.0 | -0.2, -5.5 to 5.2 | -4.2 | -8.8 to 0.5 |
| Diastolic Blood Pressure (mmHg) | **Baseline** | 81.7±0.6 |  | 80.5±1.2 |  | 1.2 | -1.5 to 4.0 |
|  | **1 year** | 78.1±0.7 | -3.6, -5.4 to –1.8 * | 81.3±1.2 | 0.8, -2.3 to 3.9 | -3.2 | -5.9 to –0.4 |
|  | **2 years** | 78.5±0.6 | -3.2, -4.9 to –1.4 * | 81.1±1.2 | 0.7, -2.5 to 3.8 | -2.6 | -5.3 to 0.1 |
| non-HDL (mg/dL) | **Baseline** | 140.5±3.4 |  | 136.1±6.2 |  | 4.4 | -9.7 to 18.6 |
|  | **1 year** | 143.1±3.5 | 2.6, -6.9 to 12.0 | 133.4±6.7 | -2.7, -19.9 to 14.5 | 9.7 | -5.3 to 24.8 |
|  | **2 years** | 144.4±3.4 | 3.9, -5.4 to 13.2 | 131.0-±6.2 | -5.1, -21.6 to 11.5 | 13.4 | -0.7 to 27.6 |
| Remnant Cholesterol (mg/dL) | **Baseline** | 33.5±1.0 |  | 31.1±1.9 |  | 2.5 | -1.9 to 6.8 |
|  | **1 year** | 23.7±1.1 | -9.9, -12.8 to –7.0 * | 31.8±2.1 | 0.7, -4.6 to 6.1 | -8.1 * | -12.8 to –3.5 * |
|  | **2 years** | 26.0±1.0 | -7.5, -10.4 to –4.7 * | 30.9±1.9 | -0.2, -5.3 to 4.9 | -4.9 | -9.2 to –0.5 |
| CIMT | **Baseline** | 0.68±0.01 |  | 0.69±0.02 |  | -0.01 | -0.05 to 0.03 |
|  | **1 year** | 0.68±0.01 | 0.00, -0.03 to 0.03 | 0.69±0.02 | 0.00, -0.04 to 0.05 | -0.01 | -0.05 to 0.03 |
|  | **2 years** | 0.67±0.01 | -0.01, -0.04 to 0.02 | 0.70±0.02 | 0.01, -0.04 to 0.06 | -0.03 | -0.07 to 0.01 |
| Apoproteins |  |  |  |  |  |  |  |
| Total ApoB | **Baseline** | 103.9±2.2 |  | 101.1±4.1 |  | 2.8 | -6.5 to 12.2 |
|  | **1 year** | 104.5±2.3 | 0.5, -5.7 to 6.8 | 102.3±4.4 | 1.2, -10.2 to 12.7 | 2.1 | -7.8 to 12.1 |
|  | **2 years** | 101.3±2.2 | -2.6, -8.7 to 3.6 | 98.8±4.1 | -2.3, -13.2 to 8.7 | 2.5 | -6.9 to 11.9 |
| Total ApoA1 | **Baseline** | 148.1±2.2 |  | 144.2 ±4.0 |  | 2.8 | -6.5 to 12.2 |
|  | **1 year** | 163.5±2.2 | 15.3, 9.3 to 21.4 * | 138.8±4.3 | 1.2, -10.2 to 12.7 | 2.1 | -7.8 to 12.1 |
|  | **2 years** | 164.3±2.2 | 16.2, 10.2 to 22.2 * | 137.0±4.0 | -2.3, -13.2 to 8.7 | 2.5 | -6.9 to 11.9 |
| ApoB: ApoA1 ratio | **Baseline** | 0.7±0.0 |  | 0.7±0.0 |  | 0.0 | -0.1 to 0.1 |
|  | **1 year** | 0.7±0.0 | -0.1, -0.1 to –0.0 | 0.8±0.0 | 0.0, -0.1 to 0.1 | -0.1 | -0.2 to –0.0 |
|  | **2 years** | 0.6±0.0 | -0.1, -0.1 to –0.0 Ï | 0.7±0.0 | 0.0, -0.1 to 0.1 | -0.1 | -0.2 to -0.0 |

**Abbreviations.** SE, standard error; CI, 95% confidence interval; LDL, low density lipoprotein; HDL, high-density lipoprotein; IDL, intermediate density lipoprotein; VLDL, very low-density lipoprotein; CIMT, carotid intima-media thickness.

**Note.**

Adjusted means and mean changes were obtained from an analysis using linear mixed-effects model (LMM) controlling for baseline age, sex, race, body mass index, HDL 2+3a, mid-zone, insulin use and statin use.

^a^ Variables normalized by removing the top 1% of values. Analyses were conducted excluding the top 1% values, although all cases were included using the maximum likelihood approach.

^b^ Variables normalized by natural log transformation. Non-transformed and unadjusted means, mean changes, CI and standard errors were provided in the table, but the significance level is calculated from the transformed analysis

* P<0.0015 ensures overall simultaneous significance of P<0.05 over the 33 variables using Bonferroni correction

Ï P<0.005

**Table S3.** Lipid lowering and anti-hypertensive medication use over time among completers

| Medications | Visit | Continuous Care Intervention (CCI)  n=194 | | | Usual Care (UC)  n=58 | | |
| --- | --- | --- | --- | --- | --- | --- | --- |
|  |  | **Mean (%)** | **SE** | **P-value** | **Mean (%)** | **SE** | **P-value** |
| Statin | **Baseline** | 52.6 | 3.1 | 0.25 | 53.4 | 5.3 | 0.12 |
|  | **2 yrs** | 49.0 | 3.6 |  | 65.5 | 6.3 |  |
| Other lipid lowering medications | **Baseline** | 9.3 | 1.9 | 8.0x10^-3^ | 15.5 | 4.3 | 1.0 |
|  | **2 yrs** | 5.2 | 1.6 |  | 17.2 | 5.0 |  |
| Any antihypertensive medications | **Baseline** | 76.8 | 2.6 | 1.0x10^-3^ | 63.8 | 5.1 | 0.34 |
|  | **2 yrs** | 66.5 | 3.4 |  | 70.7 | 6.0 |  |
| ACE & ARB | **Baseline** | 61.9 | 3.0 | 0.07 | 53.4 | 5.4 | 0.75 |
|  | **2 yrs** | 56.2 | 3.6 |  | 56.9 | 6.6 |  |
| Diuretics | **Baseline** | 40.7 | 3.0 | 7.0x10^-3^ | 25.9 | 4.9 | 0.23 |
|  | **2 yrs** | 31.4 | 3.3 |  | 34.5 | 6.3 |  |

**Abbreviations.** SE, standard error, ACE, angiotensin converting enzyme inhibitors; ARB, angiotensin receptor blocker.

**Note.** Other lipid lowering medications include bile acid sequestrants, fibrates, niacin, and omega-3 fatty acid ethyl esters. Changes in estimated mean proportion of medication use were analyzed using McNemar’s test.

**Table S4.** Adjusted means and changes in lipids, lipoproteins, apoproteins, blood pressure, and CIMT over time by treatment group (intent-to-treat analysis)

| Variables | Visit | Continuous Care Intervention (n=262) | | Usual Care (n=87) | | Between Group Effect | |
| --- | --- | --- | --- | --- | --- | --- | --- |
|  |  | **Mean ± SE** | **Change from baseline (Mean, CI)** | **Mean ± SE** | **Change from baseline (Mean, CI)** | **Mean**  **Difference** | **95% CI** |
| Lipids, blood pressure, and CIMT | | | | | | | |
| Total Cholesterol (mg/dL) | **Baseline** | 186.0±2.8 |  | 172.3±5.5 |  | 13.7 | 1.3 to 26.0 |
|  | **1 year** | 194.0±3.2 | 8.0, -0.2 to 16.2 | 172.9±5.9 | 0.6, -14.5 to 15.7 | 21.1^Ï^ | 7.8 to 34.4 |
|  | **2 years** | 196.0±3.4 | 10.0, 1.4 to 18.5 | 172.0±6.2 | -0.3, -16.1 to 15.4 | 24.0 * | 9.8 to 38.1* |
| LDL-C(mg/dL) | **Baseline** | 102.4 ±2.2 |  | 98.0 ±4.5 |  | 4.4 | -5.6 to 14.4 |
|  | **1 year** | 113.1 ±2.5 | 10.8, 4.2 to 17.3 * | 88.0 ±5.0 | -10.0, -22.8 to 2.8 | 25.2 * | 14.1 to 36.3 * |
|  | **2 years** | 115.0 ±2.7 | 12.7, 5.8 to 19.5 * | 90.8 ±5.1 | -7.2, -20.1 to 5.8 | 24.3 * | 12.8 to 35.8 * |
| HDL-C(mg/dL) | **Baseline** | 42.2±0.8 |  | 37.2±1.7 |  | 5.0 | 1.3 to 8.8 |
|  | **1 year** | 49.9±1.0 | 7.7, 5.2 to 10.2 * | 35.2±1.8 | -2.0, -6.6 to 2.6 | 14.7 * | 10.7 to 18.8 |
|  | **2 years** | 50.7±1.0 | 8.5, 5.9 to 11.1 * | 41.0±1.9 | 3.8, -1.0 to 8.5 | 9.7 * | 5.4 to 14.0 |
| Triglycerides (mg/dL)^b^ | **Baseline** | 197.2±9.1 |  | 282.9±45.1 |  | -85.7 | -177.2 to 5.9 |
|  | **1 year** | 148.9±10.1 | -48.3, -24.9 to –73.1 * | 314.5±61.4 | 31.6, -48.3 to 108.4 | -165.5 | -289.9 to –41.2 * |
|  | **2 years** | 153.3±10.4 | -43.9, -14.7 to –67.7 * | 209.5±18.5 | -73.4, -99.3 to 67.9 | -56.2 | -97.5 to –14.8 * |
| Systolic Blood Pressure (mmHg) | **Baseline** | 132.2±0.9 |  | 128.5±1.7 |  | 3.7 | -0.2 to 7.6 |
|  | **1 year** | 126.0±1.0 | -6.2, -8.8 to –3.6 * | 128.0±1.7 | -0.6, -5.1 to 4.0 | -1.9 | -5.9 to 2.0 |
|  | **2 years** | 125.8±1.1 | -6.4, -9.2 to –3.6 * | 129.4±2.0 | 0.8, -4.2 to 5.9 | -3.6 | -8.2 to 1.0 |
| Diastolic Blood Pressure (mmHg) | **Baseline** | 82.0±0.5 |  | 79.8±1.0 |  | 2.2 | -0.1 to 4.6 |
|  | **1 year** | 78.2±0.6 | -3.8, -5.4 to –2.3 * | 79.6±1.0 | -0.1, -2.9 to 2.6 | -1.4 | -3.8 to 0.9 |
|  | **2 years** | 78.5±0.7 | -3.5, -5.1 to –1.9 * | 81.0±1.2 | 1.2, -1.8 to 4.2 | -2.5 | -5.2 to 0.3 |
| non-HDL (mg/dL) | **Baseline** | 143.7±2.8 |  | 135.1±5.5 |  | 8.7 | -3.6 to 20.9 |
|  | **1 year** | 144.1±3.2 | 0.3, -7.8 to 8.5 | 137.7±5.8 | 2.6, -12.5 to 17.6 | 6.4 | -6.8 to 19.6 |
|  | **2 years** | 145.3±3.4 | 1.6, -6.9 to 10.1 | 131.0±6.2 | -4.1, -11.5 to 19.7 | 14.4 | 0.3 to 28.4 |
| Remnant Cholesterol (mg/dL) | **Baseline** | 34.8±0.9 |  | 33.2±1.8 |  | 1.6 | -2.5 to 5.7 |
|  | **1 year** | 26.2±1.0 | -8.7, -11.3 to –6.0 * | 34.6±2.0 | 1.4, -3.9 to 6.6 | -8.4 * | -13.0 to –3.9 * |
|  | **2 years** | 26.9±1.1 | -8.0, -10.8 to –5.2 * | 31.0±2.1 | -2.3, -7.6 to 3.0 | -4.1 | -8.8 to 0.6 |
| CIMT | **Baseline** | 0.68±0.01 |  | 0.69±0.02 |  | -0.01 | -0.05 to 0.03 |
|  | **1 year** | 0.68±0.01 | 0.00, -0.03 to 0.03 | 0.69±0.02 | 0.00, -0.04 to 0.05 | -0.01 | -0.05 to 0.03 |
|  | **2 years** | 0.67±0.01 | -0.01, -0.04 to 0.02 | 0.70±0.02 | 0.01, -0.04 to 0.06 | -0.03 | -0.07 to 0.01 |
| Apoproteins |  |  |  |  |  |  |  |
| Total ApoB | **Baseline** | 106.3±1.8 |  | 100.7±3.6 |  | 5.6 | -2.6 to 13.8 |
|  | **1 year** | 104.8±2.1 | -1.5, -6.8 to 3.9 | 102.2±3.9 | 1.5, -8.5 to 11.5 | 2.6 | -6.2 to 11.4 |
|  | **2 years** | 101.7±2.3 | -4.6, -10.2 to 1.1 | 98.7±4.1 | -2.0, -12.4 to 8.4 | 3.0 | -6.3 to 12.4 |
| Total ApoA1 | **Baseline** | 146.9±1.7 |  | 144.0±3.4 |  | 3.0 | -4.7 to 10.6 |
|  | **1 year** | 161.3±2.0 | 14.4, 9.3 to 19.5 * | 141.2±3.6 | -2.8, -12.2 to 6.7 | 20.1 * | 11.8 to 28.4 * |
|  | **2 years** | 163.2±2.7 | 16.3, 11.0 to 21.6 * | 137.4±3.9 | -6.6, -16.4 to 3.2 | 25.9 * | 17.1 to 34.6 * |
| ApoB: ApoA1 ratio | **Baseline** | 0.7±0.0 |  | 0.7±0.0 |  | 0.0 | -0.0 to 0.1 |
|  | **1 year** | 0.7±0.0 | -0.1, -0.1 to –0.0 Ï | 0.7±0.0 | 0.0, -0.1 to 0.1 | -0.1 | -0.1 to 0.0 |
|  | **2 years** | 0.7±0.0 | -0.1, -0.1 to –0.1 * | 0.7±0.0 | 0.0, -0.1 to 0.1 | -0.1 | -0.2 to –0.0 |
| Lipoproteins | | | | | | | |
| Total VLDL (nmol/L) | **Baseline** | 144.8±3.0 |  | 131.6±6.0 |  | 13.2 | -0.3 to 26.6 |
|  | **1 year** | 137.9±3.4 | -6.9, -15.8 to 2.0 | 133.6±6.2 | 2.0, -14.3 to 18.4 | 4.2 | -10.0 to 18.4 |
|  | **2 years** | 132.6±4.0 | -12.2, -22.0 to –2.5 | 132.2±6.8 | 0.6, -16.7 to 17.8 | 0.4 | -15.3 to 16.0 |
| VLDL Large (nmol/L) | **Baseline** | 24.4±0.8 |  | 22.2±1.5 |  | 2.3 | -1.2 to 5.7 |
|  | **1 year** | 19.9±0.9 | -4.5, -6.8 to –2.2 * | 23.6±1.6 | 1.4, -2.7 to 5.6 | -3.6 | -7.3 to –0.0 |
|  | **2 years** | 20.4±1.0 | -4.0, -6.5 to –1.5 Ï | 22.2±1.7 | 0.0, -4.4 to 4.4 | -1.7 | -5.7 to 2.2 |
| VLDL Medium (nmol/L) | **Baseline** | 58.9±1.4 |  | 54.6±2.7 |  | 4.3 | -1.7 to 10.4 |
|  | **1 year** | 53.7±1.6 | -5.2, -9.2 to –1.2 | 55.6±2.8 | 1.0, -6.4 to 8.4 | -1.8 | -8.3 to 4.6 |
|  | **2 years** | 52.8±1.8 | -6.1, -10.5 to –1.7 | 55.0±3.1 | 0.5, -7.3 to 8.2 | -2.2 | -9.3 to 4.8 |
| VLDL small (nmol/L) | **Baseline** | 61.4±1.2 |  | 54.9±2.3 |  | 6.6 | 1.4 to 11.7 |
|  | **1 year** | 64.2±1.3 | 2.8, -0.6 to 6.2 | 54.5±2.4 | -0.4, -6.7 to 5.9 | 9.7 * | 4.3 to 15.2 * |
|  | **2 years** | 59.4±1.5 | -2.0, -5.8 to 1.6 | 55.0±2.6 | 0.1, -6.5 to 6.7 | 4.4 | -1.6 to 10.4 |
| Total IDL (nmol/L) | **Baseline** | 268.4±4.7 |  | 238.3±9.2 |  | 30.1 | 9.4 to 50.8 |
|  | **1 year** | 294.2±5.3 | 25.8, 12.1 to 39.5 * | 238.4±9.6 | 0.1, -25.0 to 25.2 | 55.8 * | 34.0 to 77.6 |
|  | **2 years** | 293.0±6.1 | 24.7, 9.7 to 39.6 * | 242.1±10.4 | 3.8, -22.6 to 30.2 | 51.0 * | 27.0 to 75.0 |
| IDL 1 (nmol/L) | **Baseline** | 141.4±2.5 |  | 127.3±5.0 |  | 14.1 | 3.0 to 25.2 |
|  | **1 year** | 141.7±2.8 | 0.3, -7.1 to 7.6 | 127.1±5.2 | -0.1, -13.6 to 13.4 | 14.5 | 2.8 to 26.3 |
|  | **2 years** | 136.9±3.3 | -4.5, -12.5 to 3.6 | 127.6±5.6 | 0.3, -13.9 to 14.6 | 9.3 | -3.6 to 22.2 |
| IDL 2 (nmol/L) | **Baseline** | 127.0±2.9 |  | 111.0±5.7 |  | 16.0 | 3.3 to 28.7 |
|  | **1 year** | 152.5±3.2 | 25.5, 17.1 to 33.9 * | 111.2±5.9 | 0.2, -15.2 to 15.6 | 41.3 * | 27.9 to 54.7 * |
|  | **2 years** | 156.1±3.7 | 29.1, 19.9 to 38.3 * | 114.4±6.4 | 3.4, -12.8 to 19.7 | 41.7 * | 26.9 to 56.4 * |
| Total LDL (nmol/L) | **Baseline** | 1038.4±16.5 |  | 1014.0±33.9 |  | 24.4 | -50.8 to 99.6 |
|  | **1 year** | 994.0±18.8 | -44.4, -93.0 to 4.2 | 993.7±34.9 | -20.3, -112.8 to 92.2 | 0.3 | -78.5 to 79.1 |
|  | **2 years** | 1010.3±21.5 | -28.1, -81.0 to 24.7 | 974.3±37.9 | -39.7, -136.9 to 57.4 | 36.0 | -50.6 to 122.5 |
| LDL I (nmol/L) | **Baseline** | 167.7±4.6 |  | 150.0±9.1 |  | 17.6 | -2.7 to 37.9 |
|  | **1 year** | 206.5±5.2 | 38.9, 25.4 to 52.3 * | 142.2±9.4 | -7.9, -32.5 to 16.8 | 64.3 * | 42.9 to 85.8 * |
|  | **2 years** | 211.3±6.0 | 43.6, 28.9 to 58.3 * | 152.0±10.3 | 1.9, -24.1 to 27.9 | 59.3 * | 35.7 to 83.0 * |
| LDL IIa (nmol/L) | **Baseline** | 142.2±3.7 |  | 132.6±7.3 |  | 9.6 | -6.8 to 25.9 |
|  | **1 year** | 153.0±4.2 | 10.9, 0.1 to 21.7 | 122.5±7.6 | -10.1, -30.0 to 9.7 | 30.6 * | 13.3 to 47.8 * |
|  | **2 years** | 156.9±4.8 | 14.7, 2.9 to 26.5 | 127.4±8.2 | -5.2, -26.1 to 15.6 | 29.5 Ï | 10.5 to 48.5 Ï |
| LDL IIb (nmol/L) | **Baseline** | 182.3±4.4 |  | 174.4±8.7 |  | 7.9 | -11.7 to 27.5 |
|  | **1 year** | 169.8±5.0 | -12.4, -25.4 to 0.5 | 163.8±9.1 | -10.6, -34.4 to 13.2 | 6.1 | -14.6 to 26.8 |
|  | **2 years** | 176.4±5.8 | -5.9, -20.1 to 8.3 | 168.6±9.9 | -5.7, -30.8 to 19.4 | 7.7 | -15.1 to 30.5 |
| LDL IIIa (nmol/L) | **Baseline** | 198.0±5.8 |  | 177.7±11.5 |  | 20.3 | -5.4 to 46.0 |
|  | **1 year** | 159.3±6.6 | -38.8, -55.8 to 21.8 * | 188.3±11.9 | 10.6, -20.6 to 41.8 | -29.0 | -56.1 to –1.9 |
|  | **2 years** | 162.7±7.6 | -35.4, -53.9 to –16.8 * | 191.8±13.0 | 14.1, -18.8 to 47.0 | -29.1 | -59.0 to 0.8 |
| LDL IIIb (nmol/L) | **Baseline** | 91.2±3.5 |  | 83.5±7.0 |  | 7.7 | -7.9 to 23.3 |
|  | **1 year** | 69.2±4.0 | -22.0, -32.3 to –11.7 * | 98.0±7.2 | 14.5, -4.4 to 33.4 | -28.8 * | -45.3 to –12.3 * |
|  | **2 years** | 68.0±4.6 | -23.1, -34.4 to –11.8 * | 96.2±7.9 | 12.7, -7.2 to 32.7 | -28.1 Ï | -46.3 to –10.0 Ï |
| LDL IVa (nmol/L)^a^ | **Baseline** | 92.6±2.7 |  | 93.8±5.5 |  | -1.2 | -13.4 to 11.0 |
|  | **1 year** | 80.0±3.1 | -12.6, -20.6 to –4.7 Ï | 104.2±5.7 | 10.4, -4.5 to 25.2 | -24.2 * | -37.0 to –11.4* |
|  | **2 years** | 78.6±3.5 | -14.0, -22.7 to –5.3 Ï | 91.5±6.3 | -2.3, -18.0 to 13.4 | -12.9 | -27.1 to 1.2 |
| LDL IVb (nmol/L)^a^ | **Baseline** | 79.5±1.5 |  | 82.5±3.0 |  | -3.1 | -9.6 to 3.5 |
|  | **1 year** | 74.6±1.7 | -4.9, -9.2 to –0.6 | 83.8±3.0 | 1.3, -6.8 to 9.3 | -9.2 | -16.1 to –2.3 |
|  | **2 years** | 74.0±1.9 | -5.5, -10.1 to –0.8 | 83.9±3.3 | 1.3, -7.0 to 9.7 | -9.9 | -17.4 to –2.3 |
| LDL IVc (nmol/L)^a^ | **Baseline** | 89.6±1.0 |  | 90.1±2.0 |  | -0.5 | -5.0 to 3.9 |
|  | **1 year** | 85.9±1.1 | -3.7, -6.6 to –0.8 | 89.0±2.1 | -1.1, -6.6 to 4.3 | -3.1 | -7.8 to 1.6 |
|  | **2 years** | 84.5±1.3 | -5.1, -8.2 to –1.9 Ï | 86.7±2.2 | -3.5, -9.1 to 2.2 | -2.2 | -7.3 to 3.0 |
| Mid-zone (nmol/L) | **Baseline** | 880.7±6.2 |  | 898.7±12.3 |  | -18.0 | -45.6 to 9.6 |
|  | **1 year** | 839.2±7.1 | -41.5, -59.8 to –23.2 * | 889.1±12.8 | -9.6, -43.1 to 24.0 | -49.9 * | -79.1 to –20.7 * |
|  | **2 years** | 820.5±8.2 | -60.2, -80.2 to –40.3 * | 878.7±14.0 | -20.0, -55.4 to 15.3 | -58.2 * | -90.3 to –26.1 * |
| Total HDL (µmol/L) | **Baseline** | 23.2±0.2 |  | 24.0±0.3 |  | -0.7 | -1.4 to 0.0 |
|  | **1 year** | 23.1±0.2 | -0.1, -0.5 to 0.4 | 23.5±0.3 | -0.4, -1.3 to 0.4 | -0.4 | -1.1 to 0.3 |
|  | **2 years** | 23.2±0.2 | 0.0, -0.5 to 0.5 | 23.9±0.4 | -0.1, -0.9 to 0.8 | -0.7 | -1.5 to 0.1 |
| HDL 2b (µmol/L) | **Baseline** | 6.0±0.1 |  | 6.0±0.1 |  | 0.0 | -0.3 to 0.2 |
|  | **1 year** | 6.3±0.1 | 0.3, 0.1 to 0.5 Ï | 6.0±0.1 | 0.0, -0.4 to 0.3 | 0.3 | 0.0 to 0.6 |
|  | **2 years** | 6.3±0.1 | 0.3, 0.1 to 0.5 | 6.2±0.1 | 0.1, -0.2 to 0.5 | -0.1 | -0.3 to 0.4 |
| HDL 2a+3 (µmol/L) | **Baseline** | 17.2±0.1 |  | 17.9±0.2 |  | -0.7 | -1.2 to –0.2 |
|  | **1 year** | 16.9±0.1 | -0.4, -0.7 to –0.0 | 17.5±0.2 | -0.4, -1.0 to 0.2 | -0.7 | -1.2 to –0.1 |
|  | **2 years** | 17.0±0.2 | -0.3, -0.7 to 0.1 | 17.7±0.3 | -0.2, -0.9 to 0.5 | -0.8 | -1.4 to –0.2 |
| LDL Peak Diameters (Å) | **Baseline** | 215.3±0.4 |  | 214.7±0.7 |  | 0.7 | -1.0 to 2.3 |
|  | **1 year** | 219.2±0.4 | 3.9, 2.8 to 5.0 * | 214.7±0.8 | 0.0, -2.0 to 2.0 | 4.6 * | 2.8 to 6.3 * |
|  | **2 years** | 219.3±0.5 | 4.0, 2.8 to 5.2 * | 215.9±0.8 | 1.2, -0.9 to 3.4 | 3.4 * | 1.5 to 5.4 * |

**Abbreviations.** SE, standard error; CI, 95% confidence interval; LDL, low density lipoprotein; HDL, high-density lipoprotein; IDL, intermediate density lipoprotein; VLDL, very low-density lipoprotein; CIMT, carotid intima-media thickness.

**Note.**

Adjusted means and mean changes were obtained from an intent-to-treat analysis using linear mixed-effects model (LMM) controlling for baseline age, sex, race, body mass index, HDL 2+3a, mid-zone, insulin use and statin use. A maximum likelihood-based approach was used to estimate missing data.

^a^ Variables normalized by removing the top 1% of values. Analyses were conducted excluding the top 1% values, although all cases were included using the maximum likelihood approach.

^b^ Variables normalized by natural log transformation. Non-transformed and unadjusted means, mean changes, CI and standard errors were provided in the table, but the significance level is calculated from the transformed analysis

* P<0.0015 Bonferroni corrected significance value over 33 variables with an overall simultaneous significance of P<0.05

Ï P<0.005

**Table S5.** Principal Components and Respective Loading of Each Lipoprotein/Lipid from Baseline and Two-year Follow-up Data

| Variables | Continuous Care Intervention | | | | | | | Usual Care | | | | | |
| --- | --- | --- | --- | --- | --- | --- | --- | --- | --- | --- | --- | --- | --- |
|  | **Baseline** | | | **Two-year Follow-up** | | | | **Baseline** | | | **Two-year Follow-up** | | |
|  | **PC1** | **PC2** | **PC3** | **PC2** | **PC1a** | **PC1b** | **PC3** | **PC1** | **PC2** | **PC3** | **PC1** | **PC2** | **PC3** |
| HDL 2a+3 | 0.322 | 0.402 | **0.804** | 0.404 | **0.47** | 0.348 | **0.634** | 0.442 | 0.367 | **0.697** | 0.05 | 0.448 | **0.668** |
| HDL 2b | -0.04 | 0.275 | **0.870** | 0.171 | -0.079 | 0.104 | **0.918** | 0.302 | 0.374 | **0.888** | -0.336 | 0.432 | **0.682** |
| Mid-zone | 0.367 | 0.262 | **0.821** | 0.319 | **0.463** | **0.698** | **0.476** | **0.707** | 0.324 | **0.552** | 0.297 | 0.205 | **0.837** |
| LDL IVc | **0.606** | 0.180 | **0.791** | 0.3 | **0.523** | **0.798** | **0.443** | **0.812** | 0.182 | **0.477** | **0.467** | 0.155 | **0.851** |
| LDL IVb | **0.710** | 0.027 | **0.540** | 0.091 | 0.342 | **0.874** | 0.057 | **0.771** | 0.02 | 0.288 | **0.64** | -0.153 | **0.511** |
| LDL IVa | **0.829** | -0.071 | 0.318 | -0.035 | **0.481** | **0.862** | -0.109 | **0.837** | 0.012 | 0.053 | **0.847** | -0.201 | 0.154 |
| LDL IIIb | **0.904** | 0.038 | 0.214 | 0.099 | **0.738** | **0.76** | -0.22 | **0.865** | 0.189 | -0.062 | **0.884** | -0.084 | 0.013 |
| LDL IIIa | **0.828** | 0.344 | 0.184 | 0.32 | **0.814** | **0.651** | -0.28 | **0.761** | **0.551** | -0.062 | **0.85** | 0.332 | 0.069 |
| LDL IIb | 0.362 | **0.821** | 0.172 | **0.675** | **0.682** | 0.396 | -0.235 | 0.253 | **0.885** | 0.091 | 0.41 | **0.781** | 0.212 |
| LDL IIa | -0.051 | **0.895** | 0.215 | **0.906** | 0.328 | 0.068 | 0.084 | -0.053 | **0.891** | 0.333 | -0.084 | **0.89** | 0.283 |
| LDL I | -0.259 | **0.842** | 0.364 | **0.856** | 0.02 | -0.087 | **0.512** | -0.116 | **0.806** | **0.602** | -0.359 | **0.81** | 0.295 |
| IDL 2 | -0.119 | **0.784** | **0.601** | **0.734** | 0.056 | -0.057 | **0.731** | 0.179 | **0.833** | **0.623** | -0.187 | **0.874** | 0.288 |
| IDL 1 | **0.600** | **0.79** | **0.525** | **0.852** | **0.736** | 0.353 | 0.266 | **0.629** | **0.878** | 0.332 | **0.575** | **0.782** | 0.179 |
| VLDL Small | **0.571** | **0.749** | **0.540** | **0.828** | **0.629** | 0.211 | 0.409 | **0.615** | **0.853** | 0.386 | **0.579** | **0.735** | 0.253 |
| VLDL Medium | **0.870** | 0.405 | 0.350 | **0.547** | **0.939** | **0.414** | 0.053 | **0.826** | **0.64** | 0.223 | **0.842** | 0.393 | 0.269 |
| VLDL Large | **0.897** | 0.168 | 0.250 | 0.339 | **0.948** | **0.448** | -0.102 | **0.866** | 0.424 | 0.162 | **0.843** | 0.261 | 0.332 |
| Triglycerides | **0.788** | -0.093 | 0.067 | 0.003 | **0.804** | **0.533** | -0.150 | **0.853** | 0.172 | 0.101 | **0.741** | 0.17 | 0.364 |
| LDL-C | 0.051 | **0.769** | 0.157 | **0.932** | 0.321 | 0.109 | 0.242 | 0.218 | **0.892** | 0.273 | 0.146 | **0.883** | 0.217 |
| HDL-C | **-0.574** | 0.172 | **0.432** | 0.08 | -0.451 | -0.294 | **0.783** | **-0.437** | 0.137 | **0.617** | **-0.719** | 0.433 | 0.182 |
| % Variance Explained | 39.9 | 24.8 | 12.7 | 39.9 | 22.9 | 13.9 | 6.1 | 46.6 | 23.8 | 10.3 | 38.2 | 28.0 | 11.2 |

**Abbreviations.** PC, principal component; LDL, low density lipoprotein; HDL, high-density lipoprotein; IDL, intermediate density lipoprotein; VLDL, very low-density lipoprotein; LDL-C, low density lipoprotein cholesterol; HDL-C, high density lipoprotein cholesterol; CCI, continuous care intervention

Extraction Method: Principal Component Analysis, Rotation Method: Promax with Kaiser Normalization

Used a loading cut-off >0.4 for selecting individual lipoproteins/lipids represented in each PC (Bolded)

Note: PC are listed by % variance explained by greatest to least. CCI 2-year components PC2, PC1a and PC1b are named based on similarity in the loading lipoproteins/lipids to baseline PC2 and PC1.

**Table S6.** Estimated mean proportions and standard errors in the change of LDL phenotype patterns from baseline to two years

| Treatment Groups | Visit | LDL Pattern A | | LDL Pattern I | | LDL Pattern B | | P-value  (Baseline versus follow up) |
| --- | --- | --- | --- | --- | --- | --- | --- | --- |
|  |  | **Mean (%)** | **SE** | **Mean (%)** | **SE** | **Mean (%)** | **SE** |  |
| CCI | **Baseline** | 35.5 | 3.1 | 15.8 | 2.4 | 48.7 * | 3.3 |  |
|  | **1 year** | 63.3 | 3.6 | 15.0 Ï | 2.7 | 21.7 * | 3.1 | 1.1x10^-16^ |
|  | **2 years** | 60.7 | 4.2 | 17.0 Ï | 3.2 | 22.2 * | 3.6 | 4.2x10^-11^ |
| UC | **Baseline** | 22.2 | 5.2 | 20.6 | 5.1 | 57.1 * | 6.2 |  |
|  | **1 year** | 25.9 | 5.8 | 13.8 | 4.5 | 60.3 * | 6.4 | 0.82 |
|  | **2 years** | 29.2 | 6.6 | 25.0 | 6.3 | 45.8 * | 7.2 | 0.21 |

**Abbreviations.** SE, standard error; CCI, continuous care intervention; UC, usual care; LDL, low density lipoprotein.

**Note.**

Estimated mean proportions and standard error were obtained from the generalized estimating equation (GEE) models controlling for baseline age, sex, race, body mass index, HDL 2+3a, mid-zone, insulin use, and statin use. Only participants with available data at baseline, 1 year, and 2 years were included in the analysis.

* P<0.05 Significant difference in the proportion of LDL Pattern B versus LDL Pattern A

Ï P<0.05 Significant difference in the proportion of LDL Pattern I versus LDL Pattern A

**Table S7.** Multivariate analysis of variance (MANOVA) of lipoprotein subclasses in LDL-C hypo- versus hyper-responders

| One-way MANOVA analysis result |  |  |  |  |
| --- | --- | --- | --- | --- |
| Statistics | **Value** | **F** | **Significance** | 𝜔^2^ |
| Pillai’s Trace | 0.658 | 5.092 | **6.0x10^-6^** | 0.658 |
| Univariate analysis results | | | | |
| Variables | **Hypo-responder (n=29)**  **Mean (SD)** | **Hyper-responder (n=34)**  **Mean (SD)** | **F, p-value** | 𝜔^2^ |
| VLDL Large (nmol/L) | 15.7 (8.5) | 19.7 (9.0) | 3.4, 0.07 | 0.052 |
| VLDL Medium (nmol/L) | 42.2 (16.8) | 56.2 (17.0) | **10.7, 2.0x10^-3^** | 0.149 |
| VLDL Small (nmol/L) ^a^ | 48.2 (14.4) | 71.4 (16.1) | **38.1, 6.1x10^-8^** | 0.384 |
| IDL 1 (nmol/L) ^a^ | 111.9 (25.7) | 159.9 (31.2) | **45.0, 7.3x10^-9^** | 0.425 |
| IDL 2 (nmol/L) ^a^ | 139.2 (58.0) | 198.2 (61.9) | **20.5, 2.9x10^-5^** | 0.251 |
| LDL I (nmol/L) ^a^ | 184.9 (79.2) | 275.8 (92.8) | **22.9, 1.1x10^-5^** | 0.273 |
| LDL IIa (nmol/L) ^a^ | 127.3 (39.1) | 202.4 (62.0) | **38.8, 4.9x10^-8^** | 0.388 |
| LDL IIb (nmol/L) ^a^ | 141.8 (47.3) | 210.6 (67.8) | **22.3, 1.4x10^-5^** | 0.267 |
| LDL IIIa (nmol/L) ^a^ | 128.2 (51.8) | 173.8 (89.1) | 5.5, 0.02 | 0.083 |
| LDL IIIb (nmol/L) ^a^ | 52.4 (15.5) | 61.8 (28.2) | 1.9, 0.18 | 0.030 |
| LDL IVa(nmol/L) | 70.5 (17.5) | 69.2 (15.2) | 0.1, 0.75 | 0.002 |
| LDL IVb(nmol/L) ^a^ | 75.0 (20.9) | 70.7 (14.9) | 0.7, 0.40 | 0.012 |
| LDL IVc (nmol/L) | 84.7 (15.2) | 83.4 (12.7) | 0.1, 0.71 | 0.002 |
| Mid-zone (nmol/L) | 823.0 (148.9) | 814.6 (119.2) | 0.1, 0.80 | 0.001 |
| HDL 2b (µmol/L) ^a^ | 6.5 (1.4) | 6.2 (1.1) | 1.1, 0.29 | 0.018 |
| HDL 2a+3 (µmol/L) | 15.9 (2.0) | 17.0 (2.0) | 4.7, 0.03 | 0.072 |
| LDL Peak Diameter (Å) | 221.0 (5.7) | 221.3 (6.0) | 0.0, 0.84 | 0.001 |

**Abbreviations.** M, mean; SD, standard deviation; LDL, low density lipoprotein; HDL, high-density lipoprotein; IDL, intermediate density lipoprotein; VLDL, very low-density lipoprotein.

^a^ Variables normalized by natural log transformation. Non-transformed and unadjusted means, and standard deviations were provided in the table, but the significance level is calculated from the transformed analysis.

P<0.003 Bonferroni corrected significance value over 17 variables with an overall simultaneous significance of P<0.05 (significant variables were bolded)

**Table S8.** Multivariate analysis of variance (MANOVA) of lipoprotein subclasses in ApoB hypo- versus hyper-responders

| One-way MANOVA analysis result |  |  |  |  |
| --- | --- | --- | --- | --- |
| Statistics | **Value** | **F** | **Significance** | 𝜔^2^ |
| Pillai’s Trace | 0.372 | 1.75 | 0.07 | 0.372 |
| Univariate analysis results | | | | |
| Variables | **Hypo-responder (n=36)**  **Mean (SD)** | **Hyper-responder (n=32) Mean (SD)** | **F, p-value** | 𝜔^2^ |
| VLDL Large (nmol/L) a | 18.8 (10.2) | 21.9 (13.3) | 0.63, 0.43 | 0.009 |
| VLDL Medium (nmol/L) a | 48.8 (19.0) | 56.9 (21.6) | 2.37, 0.13 | 0.035 |
| VLDL Small (nmol/L) ^a^ | 53.9 (17.6) | 66.5 (16.7) | **10.81, 2.0x10^-3^** | 0.141 |
| IDL 1 (nmol/L) ^a^ | 123.8 (37.3) | 152.5 (33.7) | **13.60, 4.6x10^-4^** | 0.171 |
| IDL 2 (nmol/L) ^a^ | 139.2 (58.3) | 182.4 (73.8) | 8.75, 4.0x10^-3^ | 0.117 |
| LDL I (nmol/L) ^a^ | 185.6 (82.5) | 253.0 (107.2) | 7.99, 6.0x10^-3^ | 0.108 |
| LDL IIa (nmol/L) ^a^ | 137.6 (54.0) | 186.3 (64.2) | **10.23, 2.0x10^-3^** | 0.134 |
| LDL IIb (nmol/L) ^a^ | 159.1 (73.5) | 205.4 (72.7) | 8.46, 5.0x10^-3^ | 0.114 |
| LDL IIIa (nmol/L) ^a^ | 150.3 (86.3) | 180.4 (86.8) | 3.09, 0.08 | 0.045 |
| LDL IIIb (nmol/L) ^a^ | 63.6 (37.9) | 69.3 (32.3) | 1.04, 0.31 | 0.015 |
| LDL IVa(nmol/L) a | 79.3 (36.5) | 80.6 (35.1) | 0.07, 0.79 | 0.001 |
| LDL IVb(nmol/L) ^a^ | 76.7 (22.1) | 79.0 (28.5) | 0.07, 0.79 | 0.001 |
| LDL IVc (nmol/L) a | 84.3 (16.0) | 88.1 (22.3) | 0.51, 0.48 | 0.008 |
| Mid-zone (nmol/L) | 824.6 (147.2) | 823.1 (135.8) | 0.00, 0.96 | 0.000 |
| HDL 2b (µmol/L) ^a^ | 6.2 (1.4) | 6.2 (1.2) | 0.00, 0.98 | 0.000 |
| HDL 2a+3 (µmol/L) | 16.1 (2.4) | 17.0 (2.1) | 2.66, 0.11 | 0.039 |
| LDL Peak Diameter (Å) | 219.4 (6.4) | 219.7 (7.3) | 0.03, 0.87 | 0.000 |

**Abbreviations.** M, mean; SD, standard deviation; LDL, low density lipoprotein; HDL, high-density lipoprotein; IDL, intermediate density lipoprotein; VLDL, very low-density lipoprotein

^a^ Variables normalized by natural log transformation. Non-transformed and unadjusted means, and standard deviations were provided in the table, but the significance level is calculated from the transformed analysis

P<0.003 Bonferroni corrected significance value over 17 variables with an overall simultaneous significance of P<0.05 (significant variables were bolded)

**Table S9.** Relationships between change in BMI and central abdominal fat with lipids and lipoproteins

|  | Δ BMI | | | | | | Δ Central Abdominal Fat | | | | | |
| --- | --- | --- | --- | --- | --- | --- | --- | --- | --- | --- | --- | --- |
|  | **Linear**  **Regression Analysis** | | **Multiple Regression Analysis** | | | | **Linear**  **Regression Analysis** | | **Multiple Regression Analysis** | | | |
| Dependent Variables | **r^2^** | **P-value** | **r^2^** | **β** | **t** | **P-value** | **r^2^** | **P-value** | **r^2^** | **β** | **t** | **P-value** |
| Triglycerides | 0.37 | **1.4x10^-4^** | 0.39 | 0.35 | 7.60 | **1.0x10^-5^** | 0.38 | **1.7x10^-5^** | 0.39 | -0.18 | -0.95 | 0.35 |
| HDL-C | 0.52 | **6.0x10^-6^** | 0.55 | -0.15 | -0.96 | 0.34 | 0.56 | **1.8x10^-8^** | 0.55 | -0.34 | -5.29 | **6.3x10^-7^** |
| VLDL-Large | 0.28 | **0.02** | 0.26 | 0.19 | 2.12 | **0.04** | 0.27 | **0.03** | 0.26 | -0.19 | -0.90 | 0.37 |
| IDL-2 | 0.34 | **0.01** | 0.34 | 0.22 | 1.07 | 0.29 | 0.36 | **4.9x10^-11^** | 0.34 | -0.25 | -2.95 | **4.0x10^-3^** |
| Mid-zone | 0.46 | 0.09 | 0.44 | 0.13 | 1.68 | 0.10 | 0.46 | **0.03** | 0.44 | 0.12 | 1.61 | 0.11 |
| LDL IVa | 0.19 | 0.30 | 0.19 | 0.15 | 1.60 | 0.11 | 0.21 | **0.04** | 0.19 | 0.11 | 1.22 | 0.23 |
| LDL IIIa | 0.31 | **9.0x10^-3^** | 0.32 | 0.26 | 3.09 | **3.0x10^-3^** | 0.33 | **6.0x10^-3^** | 0.32 | -0.00 | -0.01 | 1.0 |
| LDL IIIb | 0.18 | 0.06 | 0.21 | 0.22 | 2.39 | **0.02** | 0.24 | **0.01** | 0.21 | -0.10 | -0.45 | 0.66 |
| LDL IIb | 0.25 | **0.01** | 0.23 | 0.23 | 2.52 | **0.01** | 0.24 | **0.04** | 0.23 | -0.01 | -0.02 | 0.98 |
| LDL I | 0.24 | 0.18 | 0.20 | -0.10 | -1.13 | 0.26 | 0.28 | **0.04** | 0.20 | -0.16 | -1.74 | 0.08 |
| HDL 2b | 0.29 | **2.0x10^-3^** | 0.29 | 0.05 | 0.24 | 0.81 | 0.34 | **4.1x10^-4^** | 0.29 | -0.30 | -3.54 | **1.0x10^-3^** |

**Abbreviations.** BMI, body mass index; Δ, delta; LDL, low density lipoprotein; HDL, high-density lipoprotein; IDL, intermediate density lipoprotein; VLDL, very low-density lipoprotein

Note. All regression analyses were adjusted with baseline value of each analyzed dependent variable.

* P<0.05 Significant difference (Bolded)

**Table S10.** Association between frequency of participants reporting BHB ≥ 0.5mM with change in lipids and lipoproteins from baseline to 2 years

| Variables |  | | | |
| --- | --- | --- | --- | --- |
|  | **Beta** | **SE** | **P-value** | **Incidence Rate Ratio (IRR)** |
| Δ Triglycerides | -0.001 | 0.001 | 0.01 | 0.999 |
| Δ HDL-cholesterol | 0.021 | 0.007 | 3.0x10^-3^ | 1.021 |
| Δ IDL-2 | 0.004 | 0.002 | 8.0x10^-3^ | 1.004 |
| Δ Mid-zone | -0.002 | 0.001 | 2.0x10^-3^ | 0.998 |
| Δ LDL I | 0.002 | 0.001 | 0.01 | 1.002 |

**Abbreviations.** SE, standard error**;** Δ, delta; LDL, low density lipoprotein; HDL, high-density lipoprotein; IDL, intermediate density lipoprotein

* P<0.05 Significant difference

**Table S11**. LDL phenotype conversions and their associations with frequency of participants reporting BHB ≥ 0.5mM from baseline to 2 years

| **Among those classified as “A” and “B” at baseline and 2 years** | | | | | | |
| --- | --- | --- | --- | --- | --- | --- |
| **Baseline** | **2 Years** | **CCI** | **UC** | **Beta** | **Incidence Rate Ratio (IRR)** | **P-value** |
| **A** | **A** | 44 (93.6%) | 8 (78.7%) | -0.37 | 0.69 | 0.55 |
|  | **B** | 3 (6.4%) | 3 (27.3%) |  |  |  |
| **B** | **A** | 25 (48.1%) | 4 (18.2%) | -0.98 | 0.37 | 4.2x10^-4^ |
|  | **B** | 27 (51.9%) | 18 (81.8%) |  |  |  |
| **Among those classified as “A” and “Both B and I included as B” at baseline and 2 years** | | | | | | |
| **A** | **A** | 44 (86.3%) | 8 (50.0%) | -0.22 | 0.80 | 0.59 |
|  | **B** | 7 (13.7%) | 8 (50.0%) |  |  |  |
| **B** | **A** | 40 (45.5%) | 8 (22.2%) | -0.60 | 0.55 | 0.005 |
|  |  |  |  |  |  |  |
|  | **B** | 48 (54.5%) | 28 (77.8%) |  |  |  |

**Abbreviations.** CCI, continuous care intervention; UC, usual care

**Note:**

Among those classified as “B” at baseline, the log count of the number of times participants reported a BHB value ≥ 0.5mM decreases by 0.98 in those remained as phenotype B versus to those who shifted their phenotype from B to A at 2 years. The incidence rate for those remaining as phenotype B is 0.37 times those shifting from phenotype B to A when the number of times the participants reported a BHB of ≥ 0.5mmol/L increases.

Among those classified as “Both B and I included as B” at baseline, the log count of the number of times participants reported a BHB value ≥ 0.5mM decreases by 0.60 in those remained as phenotype B versus to those who shifted their phenotype from B to A at 2 years. The incidence rate for those remaining as phenotype B is 0.55 times those shifting from phenotype B to A when the number of times the participants reported a BHB of ≥ 0.5mmol/L increases.

* P<0.05 Significant difference
